# Mechanical slowing-down of cytoplasmic diffusion allows *in vivo* counting of proteins in individual cells

**DOI:** 10.1038/ncomms11641

**Published:** 2016-05-18

**Authors:** Burak Okumus, Dirk Landgraf, Ghee Chuan Lai, Somenath Bakhsi, Juan Carlos Arias-Castro, Sadik Yildiz, Dann Huh, Raul Fernandez-Lopez, Celeste N. Peterson, Erdal Toprak, Meriem El Karoui, Johan Paulsson

**Affiliations:** 1 Department of Systems Biology, Harvard Medical School, Boston, Massachusetts 02115, USA; 2 Department of Physics, Universidad de los Andes, Bogota 4976-12340, Colombia; 3 Department of Biology, Suffolk University, Boston, Massachusetts 02108, USA

## Abstract

Many key regulatory proteins in bacteria are present in too low numbers to be detected with conventional methods, which poses a particular challenge for single-cell analyses because such proteins can contribute greatly to phenotypic heterogeneity. Here we develop a microfluidics-based platform that enables single-molecule counting of low-abundance proteins by mechanically slowing-down their diffusion within the cytoplasm of live *Escherichia coli* (*E. coli*) cells. Our technique also allows for automated microscopy at high throughput with minimal perturbation to native physiology, as well as viable enrichment/retrieval. We illustrate the method by analysing the control of the master regulator of the *E. coli* stress response, RpoS, by its adapter protein, SprE (RssB). Quantification of SprE numbers shows that though SprE is necessary for RpoS degradation, it is expressed at levels as low as 3–4 molecules per average cell cycle, and fluctuations in SprE are approximately Poisson distributed during exponential phase with no sign of bursting.

## Abstract (web summary)

Several proteins are expressed at too low abundance in the *Escherichia coli* (*E. coli*) proteome to be detected by standard methods. Here, the authors create a microfluidics-based platform enabling single-molecule counting of low-abundance proteins by mechanically slowing-down their diffusion in live *E. coli*.

## Graphical abstract

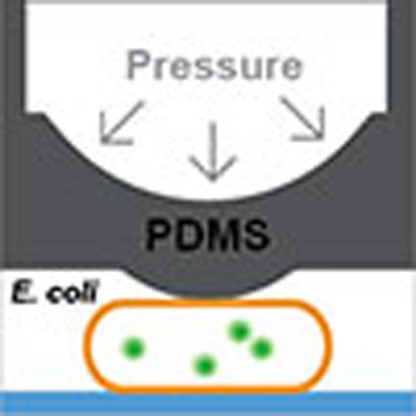

A surge of single-cell fluorescence studies has shown that genetically identical cells residing within the same environment can display extensive cell-to-cell variability in the expression levels of various proteins^1^,^2^,^3^. A substantial challenge when analysing these phenomena is that the heterogeneity typically originates in reactions involving low-abundance components, while only the high-abundance components that indirectly respond to the heterogeneity are relatively straightforward to measure. For example, many of the key regulatory proteins in *Escherichia coli* (*E. coli*) are present in such few copies—recent studies suggest that at least 10% of the proteins in *E. coli* are present in <10 copies per cell^4^,^5^—that fluorescent protein (FP) fusions are difficult to detect over the cellular auto-fluorescence^6^. Furthermore, when fluorescent levels are detectable, they are typically quantified in terms of total fluorescence and reported in arbitrary units^7^. Quantifying the total fluorescence intensity rather than counting separate copies can also introduce measurement errors, as problems with, for example, uneven excitation or detection becomes hard to separate from actual cell heterogeneity. Finally, fluctuations in protein abundances are easier to analyse mathematically when absolute numbers are known^7^,^8^. The ability to count low-abundance proteins in individual cells would thus substantially help analyse single-cell dynamics.

A recent study^4^ quantified levels of low-abundance FPs by deconvoluting the cellular autofluorescence distribution from that of the total fluorescence, which was measured separately. Though the variation in autofluorescence makes it impossible to infer the FP fluorescence in any particular single cell, that approach can still estimate the distribution over the population of cells, at least in arbitrary units of fluorescence. The challenge is that for low copy proteins, where the FP signal is a small fraction of the total, this procedure essentially infers a small quantity by taking the difference between two relatively large quantities, and is thus exceedingly sensitive to measurement errors due to imaging, growth conditions or differences in cell size.

A potentially less error-prone approach is to directly count spatially separate molecules. One early technique used single-cell capture and lysis, followed by downstream binding to antibodies to detect single protein copies^9^. Fine tuning allowed ∼60% of the molecules to be detected, but only for high-abundance proteins: the lowest abundance detected was ∼600 proteins per cell, and it was estimated that any protein present in <10 copies would fall entirely under the detection limit^9^. Quantifying protein abundances *in vivo* by microscopy could help improve detection, but the challenge is that individual proteins diffuse rapidly and appear smeared for typical exposure times. Several approaches have been used to address this problem. Chemical fixation can be used to immobilize and detect single proteins via standard total internal reflection fluorescence (TIRF)^10^,^11^ microscopy or super-resolution methods^12^ but at the expense of substantial denaturation of FPs^4^ and an increase in the cellular autofluorescence^13^. Although super-resolution methods can be used to infer stoichiometries^14^, an accurate enumeration of the protein-of-interest (POI) remains challenging because the FPs used for super-resolution imaging exhibit complicated photo-physics and suffer from a low yield of conversion into the fluorescently detectable state^15^. Otherwise cytoplasmic FPs have also been targeted to the cell membrane^16^ to slow down the diffusion, at the cost of disrupting the function of the POI. To address this issue a cotranslationally cleavable linker was added between the membrane-targeted FP and the POI^17^, but even if that could be made to work with high accuracy, the method is limited to counting proteins produced within a certain time window. All these different methods further face the challenges that the shallow depth of focus of high numerical aperture objectives is typically smaller than the height of even *E. coli* cells, making it difficult to detect all copies of the POI in a cell, and that the fluorescence of a single FP can still be difficult to separate from the cellular auto-fluorescence.

Statistical throughput can also be almost as important as resolution in single-cell studies. Interrogating large numbers of cells is not only necessary to ensure that observed differences are statistically significant^18^, but is useful for determining distributions more accurately^19^ as well as for detecting rare phenotypes^20^. High sampling further permits binning of data, where cells are grouped for instance according to their size or gene expression levels before analysing other properties. Such analysis can greatly facilitate interpretations, but the number of distinct bins increases exponentially with the number of properties measured and thus sample sizes quickly become limiting. The ability to count single molecules in single cells with a significant throughput is therefore a key requirement for analysing low-copy number protein fluctuations and the resultant cell-to-cell heterogeneity.

Here we use a simple microfluidic platform (MACS: microfluidics-assisted cell screening) to mechanically compress cells in a controlled manner. This causes diffusional slowing-down of cytoplasmic molecules without loss of fluorescence in *E. coli*, and thus enables detection of single molecules on a standard TIRF microscope set-up. The resultant flattening of the pressed cells also reduces local autofluorescence, separates the molecules spatially, and makes it easier to keep all copies within the objective depth of focus. Moreover, MACS provides automation with high throughput while growing cells in conventional liquid culture until just before the moment of imaging, and makes it possible to retrieve/enrich rare cells. To illustrate the capabilities of this technique, we applied MACS to study the control of RpoS, the master regulator of stress response in *E. coli*, by the low abundance adapter protein, SprE (RssB). Though RpoS is one of the most important and well-studied proteins in *E. coli*^21^, and SprE plays an important role in controlling RpoS levels^21^, little is known about the dynamics of this circuit because SprE is present in too low numbers^21^ to be reliably detected with conventional methods.

## Results

### Description of the MACS set-up

**Figure 1:**
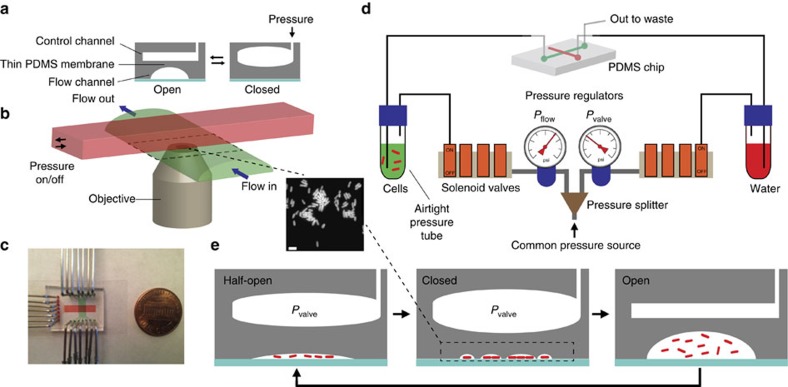
MACS set-up and workflow. (**a**) Cross-sectional schematics of the PDMS (grey) based Quake valve. A PDMS membrane separates a dead-end control channel from a dome-shaped flow channel. As the control channel is pressurized, the flexible membrane collapses onto the glass coverslip (cyan) to close the flow channel—akin to stepping on a garden hose. The flow channel rapidly re-opens as the pressure is removed. The valve can thus be actuated practically indefinitely. (**b**) Three-dimensional depiction of the valve. Control (red) and flow (green) channels run perpendicular to each other. We image underneath the region where the two channels intersect (that is, ‘valving' intersection), which is outlined by dashed lines. (**c**) Photograph of the MACS chip with control and flow channels filled with red and green dyes, respectively. (**d**) Independently controlled manual pressure regulators allow introducing pressurized air into the airtight pressure tubes (PT) to push liquid out for inducing flow of cells (green) and pressurizing the control channel (red) on the PDMS chip (via *P*_flow_ and *P*_valve_, respectively). Computer-controlled solenoid valves are used to switch *P*_flow_ and *P*_valve_, on or off. (**e**) MACS capitalizes on cycling between three distinct states of the valve (flow direction away from the page): Half-open state (*P*_flow_: on, *P*_valve_: on) is a high-resistance, low flow-rate state achieved by a certain combination of *P*_flow_ and *P*_valve_, where cells move as a monolayer underneath the PDMS membrane. When flow is stopped, the closed state (*P*_flow_: off, *P*_valve_: on) is achieved with the PDMS membrane fully sealing against the coverslip to immobilize the cells (also shown are ‘water pockets' forming around the cells) for taking fluorescence (inset, scale bar (white), 2 μm), and/or phase contrast (Supplementary Fig. 1) images of cells. Finally, the open state is executed (*P*_flow_: on, *P*_valve_: off) which is a low-resistance, high flow-rate state. The high flow rate enabled by the open state rinses the field of view and permits rapid exchange of liquid allowing for introduction of new cells that were not affected by photobleaching during imaging. Fast cycling through this sequence allows for taking multiple snapshots towards building extensive statistics (Supplementary Movie 1).

MACS uses polydimethysiloxane (PDMS)-based microfluidic on-chip valves^22^ (Fig. 1a–c), with pressure-driven flow (Fig. 1d) instead of syringe pumps to allow for easy streamlining and fast response times. Although MACS essentially exploits valve actuation to immobilize cells between a glass coverslip and a PDMS membrane similar to what was described earlier^23^,^24^, simply collapsing the valve (that is, going directly from open to closed state) yields extremely poor trapping efficiency due to the rapid displacement of liquid. Instead, MACS relies on three distinct valve states, achieved by controlling both the pressure of the valve (*P*_valve_) and the pressure driving the flow (*P*_flow_) of the cell suspension. First, the valve is closed at a certain pressure (*P*_valve_>0), while the flow is off (*P*_flow_=0) corresponding to the closed state. The pressure driving the flow is then adjusted to a level (*P*_flow_>0) that breaks the seal between PDMS and the coverslip, where cells start slipping through as a monolayer, corresponding to the half-open state (Fig. 1e). New cells are introduced, trapped and imaged by sequential cycling between half-open, closed and open valve states. Since each cycle typically takes ∼5–15 s (corresponding to ∼240–720 frames per hour), this allows automated imaging of *E. coli* cells with high throughput. As a proof-of-concept for the stability and throughput capabilities of MACS, we acquired unattended snapshots of approximately one million stationary phase *E. coli* cells in 4 h at a single valving intersection. We also imaged large numbers of cells every few minutes along the growth curve from exponential to stationary phase, revealing subtle but reproducible features of growth (Supplementary Fig. 2).

### Mechanical slowing-down of diffusion in *E. coli* cytoplasm

**Figure 2:**
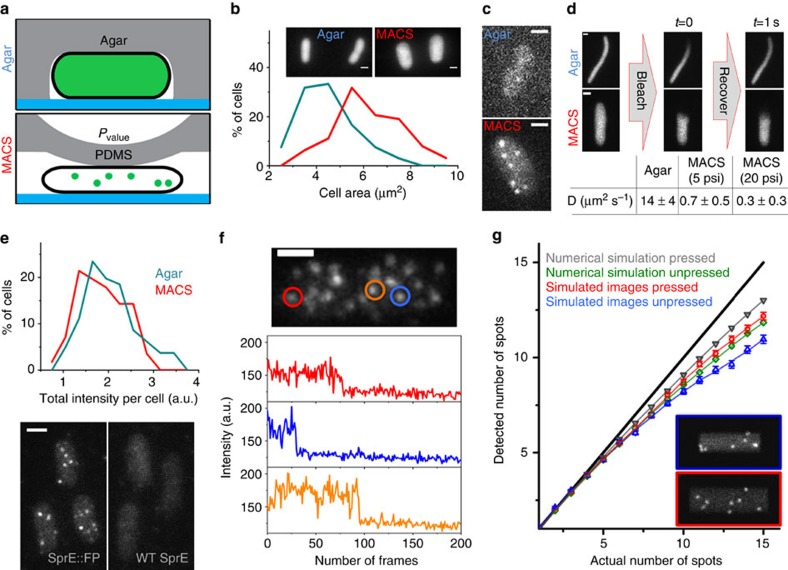
Characterization of mechanical slowing-down and single-molecule counting on MACS. (**a**–**d**) MACS induces cell deformation and mechanical slowing-down of cytoplasmic proteins. (**a**) Cartoon depicting cell flattening and appearance of discrete spots (that is, single molecules, see below) for MACS versus agar pad imaging. (**b**) In comparison with agar, cells are flattened when imaged under MACS. (**c**) SprE tagged with mNeonGreen results in discrete spots on MACS as opposed to a diffuse signal on agar. (**d**) FRAP measurements quantify the extent of mechanical slowing-down on MACS as a function of *P*_valve_ (Supplementary Fig. 4 and Supplementary Movies 4 and 5). Cells were treated with cephalexin and were thus elongated to enable FRAP measurements on agar, since FRAP occurred too rapidly to be measured otherwise. (**e**–**g**) Single-molecule counting is feasible with MACS. (**e**) Comparison of total intensities between agar and MACS imaging for the highly expressed segmentation marker (CFP) of the SprE-mNeonGreen strain suggesting that MACS does not cause signal loss. Comparing images of strains for FP-tagged versus wild-type SprE implies that the spots are specific to the mNeonGreen tagging of SprE. (**f**) Representative time traces of the SprE spots from one cell exhibiting single-step photobleaching (Supplementary Movie 6). (**g**) Undercounting due to spatial spot overlap in the cells was quantified using two independent computer simulations. First, a numerical simulation was carried out considering that the Euclidian distance between the spots be smaller than the diffraction-limited resolution (Supplementary Methods and Supplementary Fig. 6). The second simulation used computer-generated images, which were then analysed using the spot-finding code (Supplementary Methods and Supplementary Fig. 7). Inset shows two representative simulated images for unpressed (top) versus pressed (bottom) cells with an actual number of spots=8 (Supplementary Fig. 8). The results of both simulations suggest that pressing on the cells would moderately remedy undercounting. All scale bars (white) are 1 μm.

We expressed various FPs or translational fusions to FPs in *E. coli* and monitored them using HILO imaging^25^ typically with a 30-ms exposure time (see Methods). Comparing the area of cells imaged on an agar pad versus MACS revealed that cells were compressed and flattened via MACS, increasing the cell area under typically applied pressures (*P*_valve_=20 p.s.i.) on average by 72% under our conditions (Fig. 2a,b). When imaged on agar pads (without any applied pressure), cells displayed a uniform cytoplasmic signal due to the rapid diffusion of molecules (Fig. 2c and Supplementary Movie 2). In contrast, when cells were squeezed with the MACS chips with *P*_valve_=20 p.s.i., individual molecules appeared as diffraction-limited spots due to slowing-down of diffusion (Fig. 2c and Supplementary Movie 3). We speculate that this phenomenon is due to water being expelled from the cell, increasing the density of the *E. coli* cytoplasm^26^. Figure 2c shows results for a SprE-mNeonGreen translational fusion expressed from its native chromosomal locus, but similar effects were observed for other FPs tested (Supplementary Fig. 3). To further characterize this mechanical slowing-down of cytoplasmic diffusion, we carried out fluorescence recovery after photobleaching (FRAP) measurements (Fig. 2d, Supplementary Fig. 4 and Methods). For cells imaged on agar, the diffusion coefficient (*D*) of RFP mKate2 was 14±4 μm^2^ s^−1^ (±s.d., *n*=21 cells) (Fig. 2d), in agreement with the previously reported values^27^. Increasing *P*_valve_ from 5 to 20 p.s.i. decreased *D* below 1 μm^2^ s^−1^ (Fig. 2d). The average displacement of molecules within 30 ms is then ∼250 nm and single molecules of FPs should appear punctate, which is consistent with the discrete spots that we observe.

These results indicate that MACS could be used to image single cytoplasmic proteins in individual *E. coli*. It was previously suggested that proteins deform under increased molecular crowding, which could lead to denaturation of the FPs^28^. We therefore confirmed that, in contrast to other fixation methods^13^, there was no significant loss of signal as seen by comparing the total fluorescence distributions between agar pad versus MACS (Fig. 2e). We confirmed low levels of false positive detection with cells expressing no fluorescent marker, typically showing less than one spot per average cell, and under some conditions as low as 0.3 spots per average cell (Supplementary Fig. 5). Occasional complete immobilization further allowed us to detect single-step photobleaching traces (Fig. 2f), a hallmark of single-molecule detection, as expected since we use monomeric FPs and do not fuse them to oligomeric native proteins^10^.

As a final control, we compared the results from single and dual FP labelling, to determine the rate of false negatives, for example, due to incomplete FP maturation, which is particularly problematic during fast growth. Under the most challenging conditions of 25 min generation times we observed that single-labelled proteins still captured about 80% of the double-labelled proteins, perfectly consistent with the expected maturation time of 5–10 min^29^ (Supplementary Fig. 9). We further found that with dual labelling the fluorescent spots were almost twice as bright after background subtraction. Thus though maturation is a problem in all FP-based studies, the effects appear small even in the worst-case scenario for mNeonGreen and can be reduced further yet by double labelling.

The flattening of cells under MACS further helps counting in several ways. First, the ∼50% decrease in cell height (Supplementary Note 1), compared with unpressed cells, facilitates detection of the whole cytoplasmic volume, by ensuring all molecules to be within the depth of focus. Second, the spreading out of cytoplasmic volume also causes a reduction in background autofluorescence density, which improves the signal-to-noise ratio for the detected FPs. Finally, the flattening spreads the diffraction-limited spots over a larger area, reducing the probability of two spots to overlap. With any method, spot overlap can have counterintuitive effects. Specifically, cells that by chance have more molecules will have a higher fraction of overlapping spots. As opposed to most experimental errors, this artefact will narrow the observed distributions, at least the right tail (discussed more below). Our analysis suggests that for cell sizes typical of rapidly growing *E. coli*, spot overlap does not significantly interfere until levels reach above 7–8 spots per cell (Fig. 2g), and a substantial fraction of the proteome is present in lower abundances than that. However, our method can also be used to infer numbers with reasonable precision even for higher abundances; and for statistical metrics such as averages (Supplementary Fig. 10) or even distributions and variances as the data can in principle be corrected for overlap.

### Analysing the control of stress response in *E. coli*

**Figure 3:**
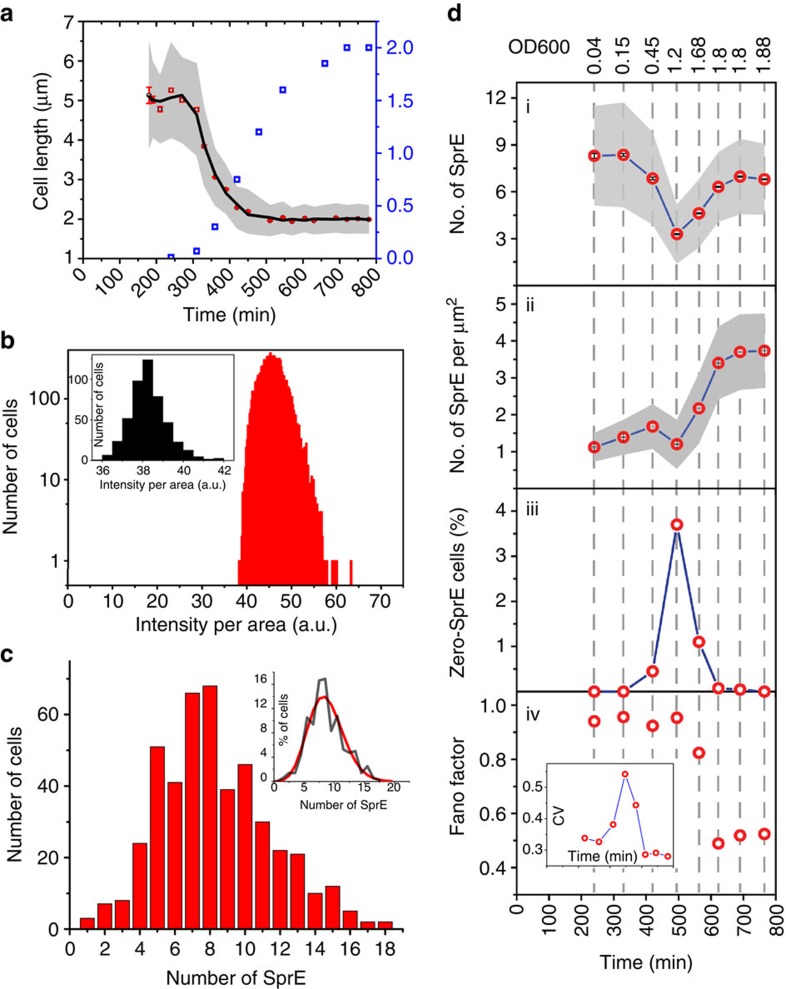
Using MACS for studying the general stress response in *E. coli*. In the presence of stress, RNAP preferentially binds to RpoS (*σ*^S^ or *σ*^38^) instead of RpoD (*σ*^70^) to direct the transcriptional program towards the expression of stress-response genes. SprE (RssB) is an adapter protein, which is involved in controlling the RpoS levels by binding to RpoS, and delivering it to the ClpXP protease for ATP-dependent degradation. (**a**) Cell-length dependence of the RpoS750-Venus strain measured along the growth curve suggest that the balanced growth (where the average cell length remains steady) is sustained for a very limited period (OD_600_ ≤0.07). Grey-shaded area is the s.d., and black line is smoothing via moving-window average. (**b**) RpoS750-Venus intensity distribution for balanced growth. Brightest cells display only twice as much intensity compared with that of the auto-fluorescence (inset). (**c**) Absolute number distribution of SprE in balanced growth (extreme cell sizes were excluded). Inset shows the normalized histogram (black) overlaid with a Poisson distribution of the same mean (red). (**d**) SprE counting at various OD_600_ allows monitoring (i) number of SprE molecules per cell, (ii) number of SprE molecules per cell normalized by the cell area as a metric for SprE concentration, (iii) fraction of cells with zero SprE molecules and (iv) Fano factor (which is equal to σ_*p*_^2^/〈*p*〉, where *σ*_*p*_ is the s.d. and 〈*p*〉 is the mean of SprE number distributions) along the growth curve of *E. coli*. Fano factor after length conditioning appears to deviate from that of Poisson distribution (Fano factor=1) after OD_600_∼1.2 most likely due to undercounting (Supplementary Fig. 13). Inset shows coefficient of variation (CV=*σ*_*p*_/〈*p*〉). Grey-shaded areas represent the s.d., and blue lines are line segments connecting data points.

To illustrate the approach, we used MACS to analyse fluctuations in the control of the master regulator of stress response in *E. coli*—the alternative sigma factor RpoS (also known as *σ*^S^ or *σ*^38^). In the presence of various stress factors such as oxidative stress, low pH, high osmolarity or nutrient limitations, RpoS replaces its vegetative counterpart RpoD to regulate the transcriptional program of *E. coli* by redirecting RNA polymerase to transcribe ∼500 genes^21^ (Fig. 3). To prevent this from occurring under non-stressful conditions, RpoS is delivered to the ClpXP protease by the adapter protein SprE, rendering RpoS one of the most short-lived proteins in *E. coli*^30^ during exponential growth. SprE has been reported to be rate-limiting for RpoS degradation in exponential phase^31^, but levels are so low as to be almost undetectable using either western blots^32^,^33^ or standard fluorescence imaging^21^. Although the activity of SprE is regulated in various ways, this raises the question of whether heterogeneity in the abundance of SprE could create heterogeneity in RpoS. Specifically, if SprE levels are low, a substantial fraction of cells may contain zero SprE copies—particularly if production is burst-like as reported for many other proteins^34^. Because RpoS is so short-lived and its levels are sensitive to SprE (Supplementary Fig. 11), it could quickly accumulate during those time windows and ensure that a subpopulation of cells is ready for the stress before it occurs.

To test whether RpoS is randomly activated in the absence of external cues, we considered the balanced growth regime where cells have fully adjusted to the growth conditions but have not yet started affecting each other by depleting available nutrients or accumulating metabolic waste^35^. In keeping with previously reported results^35^, we observed that this regime is only sustained at extremely low cell density, before OD_600_ can be reliably measured (Fig. 3a). As demonstrated above, MACS can be used to image on the order of >10^5^ cells per hour in dense culture. Here we instead exploited the automation and ability to perform imaging under native conditions to image about 10^3^ cells per hour in the very dilute balanced growth regime without concentrating cells, since the latter could trigger an RpoS response. We measured RpoS levels in a strain where a truncated version of RpoS fused with a rapidly maturing YFP (RpoS750-Venus) is present as an additional chromosomal copy. The RpoS750 truncation has been widely used to report RpoS levels and is driven by the native *rpoS* promoter^36^,^37^ but because it is so hard to confirm that fusions do not interfere with unstable low-abundance components we only use it for qualitative conclusions. We observed a rather narrow distribution of fluorescence without a single outlier displaying high RpoS750-Venus signal in ∼11,500 cells (Fig. 3b), that is, none of the cells had RpoS levels close to what is observed in stationary phase. Thus if a sub-population of cells are in a high-RpoS state in the absence of external cues—hedging the bets in case the population is suddenly exposed to stress—it appears to occur with a frequency of <10^–4^. This conclusion holds even if the FP fusion interferes with RpoS degradation in exponential phase, since artificially stabilizing RpoS would lead us to overestimating rather than underestimating levels.

We then used the single-molecule counting capabilities of MACS to determine SprE levels under the same growth conditions where SprE was tagged with mNeonGreen at its C terminus resulting in a functional fusion (Supplementary Fig. 11). Our FP of choice, mNeonGreen displays high brightness and photostability, and has a relatively short maturation time of <10 min (ref. 29). Since this is much shorter than the protein elimination rate through dilution, which is equal to the doubling time of ≥25 min under our conditions, only minor corrections are needed and we therefore report the raw data. We observed, in multiple separate experiments, that the SprE-mNeonGreen distribution had a reproducible average of 7–8 copies per cell and a standard deviation of ∼3 copies over individual cells (Fig. 3c). This low number is in agreement with the previously reported undetectable levels of SprE in early exponential phase^33^. By conditioning the data on cell size, we further observe that the distribution closely follows a Poisson in each size class (Fig. 3c), in contrast to what has been observed for most other proteins in *E. coli*, which additionally show signs of translation bursts or extrinsic noise^4^,^7^. This observation is consistent with the fact that SprE appears to be weakly translated^38^ and that the SprE mRNA is relatively short-lived^39^, compared with genes that are reported to exhibit significant bursting^34^,^40^. Phrased differently, Poisson noise is expected to increasingly dominate at lower average protein abundances, unless the low protein abundances are caused by lower mRNA numbers, which is not the case here because *sprE* is strongly transcribed^38^.

In balanced growth we did not observe a single cell with zero SprE-mNeonGreen molecules, and based on the apparent Poisson statistics the probability of such events should be on the order of 10^−4^ (or less given that a few immature FPs are missed). Because SprE-mNeonGreen is not substantially degraded, the results further suggest that cells produce ∼4–8 molecules per average cell cycle, while the Poisson statistics and absence of bursts suggest that those production events are effectively independent. Thus the few cells that temporarily have zero SprE molecules should only remain in that state for a few minutes on average, providing little time to boost RpoS levels.

We next measured SprE-mNeonGreen levels at various OD_600_ (Fig. 3d) to decipher SprE dynamics along the growth curve. Starting at an observed average of 8.3±3.2 copies per cell (±s.d., *n*=458 cells), SprE-mNeonGreen levels went through a minimum of 3.3±1.9 copies per cell (±s.d., *n*=2928 cells) in mid-exponential phase (OD_600_ ∼1.2), and then went up again to 6.7±2.3 copies per cell (±s.d., *n*=4308 cells) in early-stationary phase (OD_600_ ∼1.9). As discussed earlier, the values in early exponential phase are likely to be slightly underestimated due to incomplete FP maturation, but this problem decreases with increased cell division time. The reported dip, which has not been previously observed because even average SprE levels were experimentally unobservable, should thus be slightly more pronounced when correcting for mNeonGreen maturation time. Because the cells become substantially smaller during this interval, the SprE concentration increases about five-fold (Fig. 3d). The mechanisms causing the dip are unknown, but could reflect the fact that SprE is expressed from two promoters^41^ or that competition for the gene expression machinery is reduced after ribosomal genes are no longer expressed.

After conditioning on cell size (Supplementary Fig. 12), the distributions were again close to Poisson for most of the growth curve (Fig. 3d). In late exponential phase, the raw distributions appear even narrower than Poisson. This could in principle be explained by the fact that RpoS and SprE are thought to form a negative feedback loop in mid-exponential phase^31^,^41^. However, a more likely explanation is that the method approaches its limits since the cell size of the MC4100 strain shrinks greatly in late exponential phase. The diffraction-limited spots then inevitably overlap and the observed distributions are in fact expected if the actual distribution is Poisson (Supplementary Fig. 13). However, spot overlap has a marginal impact on the average abundance and on the left tail of the distribution, which is particularly interesting in this context. We observe virtually no cells with zero SprE copies, except close to the minimum average abundance at OD_600_ ∼1.2, where this fraction reaches as high as a few percent (Fig. 3d). However, even in that regime, the RpoS distribution does not show any substantial outliers (Supplementary Fig. 14), perhaps because the RpoS half-life then is much longer^30^ making RpoS much less responsive to brief periods with zero SprE molecules. Thus despite the fact that bet-hedging has been suggested for RpoS, and the dedicated adapter protein SprE necessary for its degradation is present in such extremely low numbers, we see no evidence for bet-hedging at the frequencies we can measure. Instead we observe that, given the low abundances, the SprE distribution is quite narrow and with less signs of bursts than observed for most other proteins in *E. coli*. We further used our set-up to show that SprE production ceases quickly on exit from stationary phase: the total numbers of SprE-mNeonGreen per cell remain virtually unchanged until cells become large enough to divide at which time they become diluted between multiple cells (Supplementary Fig. 15).

## Discussion

We show here that applying pressure to cells in a controlled manner makes it possible to count low-abundance proteins. We speculate that the diffusional slowing-down reflects increased cellular crowding effects (Supplementary Note 2) where objects as small as individual proteins experience the cytoplasm as a glassy medium. Indeed the bacterial cytoplasm has been shown to display properties of a colloidal glass for molecules larger than 30 nm (ref. 42) under normal conditions, and chemically induced osmotic compression can lead to reduction of cytoplasmic diffusion^43^ and hindrance of intracellular signalling due to overcrowding^44^. However, regardless of the underlying physical explanation, the insignificant loss of fluorescence on compression as opposed to chemical fixation (Supplementary Fig. 16) combined with the low numbers of false positives and the improved counting in flattened cells, allow for the integer counting of low-abundance proteins using a standard TIRF set-up.

In addition, MACS offers a simple and robust microfluidic platform for microscopy with high statistical power and automation. Many other microfluidic methods allow large numbers of cells to be monitored for long time windows^3^,^45^,^46^,^47^ but to our knowledge, MACS uniquely permits high-throughput imaging while cells grow in conventional liquid culture conditions until the moment of imaging. This allows a more direct comparison to the large literature based on shaking liquid cultures, not only because several processes can be affected by contact to other cells or the walls of the device, but also because microfluidic growth chambers substantially affect the age structure of the populations. For example, in liquid culture newborn cells tend to be twice as prevalent as dividing cells, whereas this is not the case in many microfluidic growth chambers where one progeny is washed away. The inner dimensions of MACS are also very flexible and can accommodate a wide range of cell sizes and shapes without any modifications: the same devices work for cells over a 100-fold range of volumes—from micrometre-sized bacteria such as *E. coli* and *Bacillus subtilis* to 10–15 μm long eukaryotic cells such as *Saccharomyces cerevisiae* and *Schizosaccharomyces pombe* (Supplementary Movies 7 and 8).

**Figure 4:**
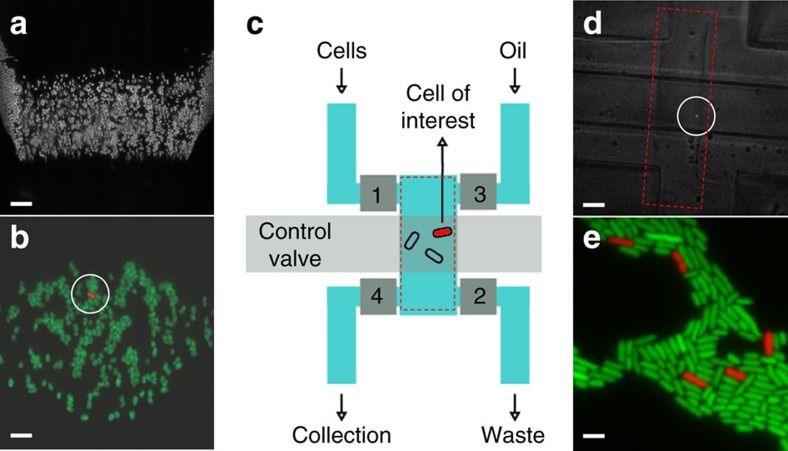
MACS allows for rapid screening and recovery/enrichment of rare cells. (**a**) Single frame of a movie (Methods, Supplementary Movie 9) of GFP-expressing *E. coli* cells flowing during the half-open state. Cells appear somewhat blurred due to their constant movement within the exposure time. Scale bar (white), 20 μm. (**b**) RFP-expressing cells, spiked in with a dilution factor of 1:100,000 (red:green), could be captured within the field of view typically in ∼3–5 min. After the red cell was immobilized (circled), snapshots in RFP and GFP fluorescence channels were taken (shown here as overlaid). Scale bar (white), 5 μm. (**c**) Minor modification of MACS enables cell retrieval (Methods, Supplementary Movie 10). (**d**) Bright-field and RFP fluorescence images are overlaid to show the captured cell of interest (circled) within the trapped volume, which is outlined by the red dashed line (control valve is open, valves 1–4 are closed). Scale bar (white) is 40 μm. (**e**) When the trapped volume was collected, grown overnight and imaged on the agar pad; the RFP-expressing cells were enriched. Counting red versus green cells suggested an enrichment factor of 10^2^ to 10^3^ (*n*=4 runs). At low cell densities this allows for the immediate retrieval of cells, and at high densities a second round is necessary to achieve 100% purity. Scale bar (white), 2 μm.

Rather than carrying out distinct cycles of cell trapping and imaging, MACS can alternatively run continuously in the half-open valve state to flow a stream of cells through the field-of-view (Fig. 4a, Methods). This can be used to detect rare phenotypes, for example where the readout is fluorescence levels above some threshold, or the presence of a spatial pattern, and then trap the identified cells for subsequent detailed imaging (Fig. 4b). Moreover, minor modifications to the design allowed us to enrich/isolate rare phenotypes by retrieval of the entrapped cells from the device (Fig. 4c–e). Taken together, we believe these features of MACS substantially extend our ability to quantify processes at the level of single molecules and in single living cells.

## Methods

### Chip fabrication and MACS properties

MACS chips were produced via soft lithography using PDMS. The base (part A) and the curing agent (part B) of a two-part silicone elastomer kit (Slygard 184, Dow Corning) were mixed at particular ratios (part A:part B) in weight to produce PDMS. The master mould for the flow channel was produced by spin-coating positive photoresist (PR) AZ10xt (AZ Electronic Materials) to a height of 10 μm on a silicon wafer. After ultra violet patterning the PR using a transparency mask (Output city), the wafer was heated for rounding the features to achieve dome-shaped channels. After rounding, the channel height becomes 8 μm. The wafer was then baked on a hotplate overnight to stabilize the positive PR. The control layer master was made by spin-coating the negative PR SU-8 2025 (MicroChem) to yield a height of 25 μm, and ultra violet patterning it using a transparency mask defining the channels. To produce the soft MACS chip, 20:1 PDMS was spin-coated on the flow channel master at 1,250 r.p.m. for 45 s to yield an ∼65-μm-thick PDMS membrane. For this condition, the minimum pressure required for closing the valve is ∼5 p.s.i. For even gentler handling of cells, if required, a thinner membrane can be made to achieve valve closing at lower pressures. For control channels, PDMS with a 5:1 ratio was poured onto the control layer master. After both masters were partially cured at 65 °C for 33 min, they were aligned and cured for another 6 h at 65 °C to achieve thermal bonding. Finally, the two-layer PDMS chip was plasma-bonded permanently against glass coverslips. Since the freshly bonded chips did not work due to altered surface properties following plasma treatment, they were kept at room temperature for at least 1 day to regain the native surface properties. For single-molecule counting experiments, the chips were kept at the 65 °C for a total of 3 days after cover glass bonding since ‘cytoplasmic slowing-down' works better with stiffer PDMS.

Closing properties of the valve depend on multiple parameters^48^. To achieve the half-open state for the 200-μm-wide control and flow channels, we typically used ∼20 p.s.i. both for *P*_valve_ and *P*_flow_, though different combinations of P_valve_ and P_flow_ also work. Compared with the full footprint of the valve (200 μm × 200 μm), cell trapping happens within a subregion (approximately, 100 μm × 50 μm), which can be varied by slight modifications of *P*_valve_ and *P*_flow_. The number of cells captured per field-of-view (FOV) also depends on the relative values of *P*_valve_ and *P*_flow_, as well as durations of the valve states. Since Quake valves can be actuated millions of times without signs of fatigue^22^, the bottleneck for long-term stability of MACS is the accumulation of debris within the valving intersection. This is problematic only during actuation of the valve and the presence of sample flow: debris does not get stuck permanently unless pressed against the surface during valve actuation and eventually gets washed away otherwise. Therefore, the intersections that remain passive do not collect debris and a neighbouring intersection can be used on demand if the actively used intersection becomes clogged. To minimize debris, we filtered all buffers and media using 0.22-μm-pore-size filters (Corning). Cells were grown in plastic tubes (BD Falcon, round-bottom) instead of glass vials to prevent crumbled glass. Sonicating the PDMS chips in isopropanol for 30 min, followed by 4 h of drying at 65 °C before bonding them to the cover glass removes PDMS crumbs that form at the inlets during hole punching^49^. In addition, prior to using the chips, flow channels were extensively rinsed with PBSA buffer (1 × PBS with 4 mg ml^−1^ BSA) to wash away debris that was stuck on the walls of the chip, as well as for passivating the channel surfaces to minimize cell sticking. We were able to keep the same chip on the microscope and use it for multiple days, everyday using a fresh flow channel.

### Microscopy

Epi-fluorescence and HILO (highly inclined and laminated optical sheet) microscopy was carried out similar to what was described previously^10^. In brief, images were collected using an Electron Multiplying CCD camera (EMCCD, iXon3 897, Andor), and OPSL lasers (Coherent) were used for HILO and FRAP measurements. The EM gain was set to 300 for single-molecule imaging. The HILO angle was adjusted using a custom-built stepper motor system. A manual flip mirror (Newfocus) allowed for switching between two different laser paths set-up for HILO and FRAP modes, respectively. For FRAP experiments, a full mirror was replaced with an 80:20 beam-splitter to allow for easy switching between a focused laser pulse and epi-fluorescence illumination. Applying a 100-ms photo-bleaching laser pulse focused on one pole of an *E. coli* cell using a mechanical shutter (Uniblitz), the fluorescence recovery was monitored via epi-fluorescence imaging. The microscope was controlled by Micro-Manager (http://www.micro-manager.org/) and custom-written MATLAB scripts. Fluorescence imaging was performed with an LED system (SOLA light engine, Lumencor) and appropriate filter cubes (Semrock): for cyan fluorescence, CFP-2432A; green fluorescence, GFP-3035B; yellow fluorescence, YFP-2427A; and red fluorescence, mCherry-A. Unless otherwise stated, we used a × 100 TIRF objective (Nikon, TIRF, numerical aperture=1.45) in combination with a × 2.5 relay lens in front of the EMCCD camera. At the expense of smaller FOV (hence lower throughput), this magnification provides a near-optimal effective pixel size (64 nm) to resolve spots and segment cell boundaries. Solid-state lasers (Coherent) in combination with proper filters (Semrock) are used for detecting single molecules (dichroic: Di01-R488, emission filters: FF01-550/88 and LP02-514RS, laser: Genesis MX 514-1000 STM OPSLaser-Diode System for mNeonGreen; dichroic: Di01-R532, emission filters: FF01-607/70 and LP03-532RS, laser: Genesis MX 532-1000 STM OPSLaser-Diode System for mEos2).

### Construction of *E. coli* strains

Strain construction is described in Supplementary Note 4. All *E. coli* strains, plasmids and primers used are listed in Supplementary Tables 2–4, respectively.

### SprE and RpoS measurements

Overnight cultures were grown in 1 × M9 salts supplemented with 10% (v/v) LB (M9+10%LB), and kept at stationary phase for a defined duration (∼16 h). After diluting the overnight cells typically in 30 ml of M9+10%LB by a factor of 10^6^, we divided the 30-ml culture into 10 falcon tubes of 3 ml each and grew cultures at 37 °C with shaking at 200 r.p.m. We used separate tubes for different time-points along the growth curve to ensure constant growth conditions for each sampling to ensure reproducibility since the RpoS levels are sensitive to aeration. Once the cells were manually transferred from the shaking flask into an airtight pressure tube (Supplementary Fig. 17), we took images for ∼15–30 min (*T*_doubling_ ∼25 min) in an automated fashion. At the end of each set of data acquisitions, we were able to clear the system off the cells completely. Ensuring that there is no carryover between different samples is vital since any cell that sticks around from earlier samplings may encounter stress, which is particularly important for studying the components of the stress response (namely, RpoS and SprE) and to minimize artefacts. To gather reasonable statistics, we pooled data from multiple samplings from OD_600_ ≤0.07. For SprE counting at high ODs, when the density of the cell culture is very high, cells tend to aggregate into large clumps under MACS, reducing the effective pressing and cytoplasmic slowing-down. Since the cell density in the FOV can be adjusted by simply changing *P*_flow_ and/or the duration of the half-open state, (Supplementary Fig. 18) this allows counting at very high cell density by minimizing the formation of cell clumps.

### Western blotting against RpoS

Western blotting was performed as previously described^10^. In brief, overnight cultures of the respective *E. coli* strains were diluted 1:100 using fresh LB medium (w/o antibiotics) and grown for 2.5 h at 37 °C with agitation (220 r.p.m.). The OD_600_ of the cultures was monitored during bacterial growth and all samples were taken when the cultures had OD_600_ readings between 0.69 and 0.79. For each sample, equal volumes of the liquid cell culture were pelleted by centrifugation (10,000*g*, 1 min) and the cell pellets were re-suspended in one-tenth of the original culture volume using a 1 × SDS loading buffer (80 mM Tris-HCl pH 6.8, 2% (w/v) SDS, 10% (v/v) glycerol, 5% (w/v) ß-mercaptoethanol, 0.01% (w/v) brophenol blue). The samples were boiled for 5 min at 95 °C. Ten microlitres of each sample was loaded on a Tris-glycine SDS–polyacrylamide gel electrophoresis containing 10% (v/v) acrylamide. The primary antibody was the monoclonal anti-RpoS antibody (Neoclone, W0009) and used at a 1:1,000 dilution. The secondary antibody was an anti-mouse horseradish peroxidase-coupled antibody (GE Healthcare, NA931), which was diluted 1:5,000 in 1 × TBST with 4% (w/v) milk powder, prior to use. The protein bands were visualized using a homemade ECL reagent and standard film.

### Characterization of MACS-induced slowing-down

For FRAP measurements, *E. coli* strains expressing cytoplasmic GFP or RFP were used. On 1,000 × dilution from an overnight culture, cells were grown at 37 °C in a shaker until the culture reached an OD_600_ of ∼0.1. Imaging of cells on agar pads was carried out as previously described^10^. To facilitate FRAP measurements on agar pads, samples were prepared by treating the cells with a final concentration of 20 μg ml^−1^ cephalexin and allowing the cells to grow for another 30 min before the measurements. We observed that slowing-down of cytoplasmic fluorescent protein molecules with MACS was more prevalent for isolated cells, presumably since a group of cells support each other against squishing. Moreover, since the closing properties of the valve are not uniform across the valve, the cells close to the edges experienced less squishing indicated by faster signal recovery. Therefore, we concentrated on the central region of the valve, and carried out FRAP on isolated cells to quantify the dependence of the diffusion coefficient on *P*_valve_. FRAP analysis for determining the diffusion coefficient was carried out using MicrobeTracker (http://microbetracker.org/) as described previously^50^. An *E. coli* strain, which constitutively expresses mEos2 from a plasmid was used for the single-molecule imaging experiments with HILO microscopy. The fraction of mEos2 molecules that happen to be spontaneously in the red state was scarce enough to allow these measurements.

### Numerical simulations for single-molecule counting

A computer simulation was developed to assess the degree of potential protein undercounting due to apparent spatial clustering of molecules caused by the ‘large' point-spread function (that is, a typical full-width at half-maximum is ∼250 nm) and the relative small size of the bacterial cell (2–6 μm^2^). Different cell sizes and geometries were compared. In short, *N* molecules were randomly placed in a virtual bacterial cell and the number of spatially resolved molecules and non-resolved clusters of molecules were calculated using Euclidian geometry and a spatial resolution of 250 nm (Supplementary Note 2 and Supplementary Figure 19). The computer code was developed and executed in MATLAB, which is available on request.

### Spot-finding analysis and simulation of EMCCD images

Determining the number of spots in single cells was achieved in two steps. First, a spot-finding software was used to detect single molecules in the entire FOV. The next step involved assigning those spots to specific cells. Therefore, we used fluorescent images of a cytoplasmic CFP as a segmentation marker to obtain an outline of the individual cells such that spots could be assigned to each cell according to their *x*–*y* coordinates. The software used for spot-finding was modified based on a previously published single-particle tracking software^51^,^52^. To summarize, each image was first computationally filtered prior to spot localization using a band-pass filter to remove high-frequency noise and low-frequency features like cellular autofluorescence signal. This process results in a smooth zero-background based image. We detected local maxima with pixel level accuracy in the image via a user-defined intensity threshold. Sub-pixel localization of the spots was then estimated from the centroids of the spots calculated using a 7 × 7 pixel square centred on the local maxima. The box size, intensity threshold, and parameters for the band-pass filter were empirically optimized to minimize false positives and false negatives in the spot detection.

As described in the main text, the small confinement volume of the *E. coli* cytoplasm imposes severe limitations for counting performance using a diffraction-limited imaging system. Since the capabilities of MACS or agar pad-based imaging may also depend on the image analysis software being used to detect the molecules, we have therefore quantified the limits of the counting performance using simulated EMCCD images that closely mimic our actual microscopy data in addition to the numerical simulations (Supplementary Note 3).

### Capturing rare phenotypes and their retrieval with MACS

For the spiking-in experiments, overnight cultures of GFP- and RFP-expressing strains inoculated from fresh bacterial re-streaks on plates were used. We found that cells grown from older-than-a-week plates display higher tendency to stick to the PDMS chip surfaces. After mixing the RFP-expressing cells with GFP-expressing cells in the pressure tube using the dilution factor of 10^5^, cells were sent through the MACS chip in the half-open valve state. Detection of the RFP-expressing cell-of-interest was achieved manually. Two inlets and two outlets on the modified design can all be controlled via on-chip valves (1–4), and allow for cell collection. Screening is carried out while valves 3 and 4 are closed, and valves 1 and 2 are open. When a cell-of-interest is captured within the FOV, cell flow is stopped. After taking detailed images, valves 1–4 are closed, and the control valve is opened to release the pressure on the cells. Subsequently, the trapped volume is sent out to collection by opening valves 3 and 4, and flowing in oil. Using an oil phase for cell collection provides precise control of the volume that is retrieved. To facilitate the collection using the oil phase, chips were treated with a commercial water repellent^49^ (Aquapel) after plasma bonding and kept at room temperature until use.

### Data availability

The data that support the findings of this study are available from the corresponding authors on request.

## Additional information

**How to cite this article**: Okumus, B. *et al.* Mechanical slowing-down of cytoplasmic diffusion allows *in vivo* counting of proteins in individual cells. *Nat. Commun.* 7:11641 doi: 10.1038/ncomms11641 (2016).

## Supplementary Material

Supplementary Information Supplementary Figures 1-19, Supplementary Tables 1-4, Supplementary Notes 1-4 and Supplementary References

Supplementary Movie 1 The movie shows three consecutive cycles of half-open, closed and open valve states. The excitation light is kept on all the time for display purposes although it is normally only turned on during the closed state to acquire snapshots when the cells are fully immobilized. As the flow is stopped to achieve the closed state (subsequent to the half-open state), liquid underneath the valve is displaced to a certain extent and the cells move - albeit transiently. Because of this brief redistribution of cells right after switching to the closed valve state, cells must be given enough time for coming to a full stop before snapshots are taken. Typically wait times are around 2-10 sec. depending on the chip, strain properties and optical density. Movie playback speed is slowed down for clarity. Effective magnification is 250×.

Supplementary Movie 2 HILO imaging of mEos2-expressing *E. coli* cells on agar pads with 30-msec exposure time. The mEos2 signal appears smeared due to the rapid diffusion of the mEos2 molecules within the cytoplasm. Nine consecutive frames of this movie are shown in Supplementary Figure 2 (top panel). Movie is in real time. Effective magnification is 250×.

Supplementary Movie 3 HILO imaging of mEos2-expressing *E. coli* cells with 30-msec exposure time on MACS with P_valve_ = 20 psi. The mEos2 molecules appear as crisp spots due to the mechanical slowing-down of diffusion. Nine consecutive frames of this movie are shown in Supplementary Figure 3 (bottom panel). Movie is in real time. Effective magnification is 250×.

Supplementary Movie 4 *E. coli* cells imaged on an agar pad after cephalexin treatment for FRAP experiments. The brief, bright spark at the 12 o'clock position is the photobleaching laser pulse. The recovery after photobleaching is fast due to rapid diffusion of molecules in the cytoplasm. Movie is in real time. Effective magnification is 150×.

Supplementary Movie 5 *E. coli* cells trapped using MACS with P_valve_ = 20 psi for FRAP experiments. The brief, bright spark at the 12 o'clock position is the photobleaching laser pulse. Compared to Supplementary Movie 4, the recovery after photobleaching is much slower due to the mechanical slowing down of molecules within the cytoplasm. Movie is in real time. Effective magnification is 150×.

Supplementary Movie 6 HILO imaging of *E. coli* cells expressing SprE-mNeonGreen with 30-msec exposure time on MACS with P_valve_ = 20 psi. The SprE-mNeonGreen molecules appear as crisp spots due to complete mechanical immobilization. Representative time traces from this movie are shown in Figure 2f. Movie is in real time. Effective magnification is 250×.

Supplementary Movie 7 MACS with *S. cerevisiae* cells. Movie is in real time. Effective magnification is 250×.

Supplementary Movie 8 MACS with *S. pombe* cells. Movie is in real time. Effective magnification is 250×.

Supplementary Movie 9 MACS run in the video mode. Cells continuously flow through the field of view as a monolayer allowing rapid interrogation of a very large number of cells. One sample frame from this movie is shown in Figure 4a in the main text. Movie is in real time. Effective magnification is 60×.

Supplementary Movie 10 Imaging of the collection outlet showing the retrieval of cells in the trapped volume using an oil phase.
