# Corrigendum: Mechanical slowing-down of cytoplasmic diffusion allows *in vivo* counting of proteins in individual cells

**DOI:** 10.1038/ncomms12130

**Published:** 2016-06-24

**Authors:** Burak Okumus, Dirk Landgraf, Ghee Chuan Lai, Somenath Bakshi, Juan Carlos Arias-Castro, Sadik Yildiz, Dann Huh, Raul Fernandez-Lopez, Celeste N. Peterson, Erdal Toprak, Meriem El Karoui, Johan Paulsson

Nature Communications 7: Article number: 1164110.1038/ncomms11641 (2016); Published: 05182016; Updated: 06242016.

The original version of this Article contained an error in the spelling of the author Somenath Bakshi, which was incorrectly given as Somenath Bakhsi. This has now been corrected in both the PDF and HTML versions of the Article.

