# Supplementary material for: Mechanical slowing-down of cytoplasmic diffusion allows in vivo counting of proteins in individual cells
Source: Nat Commun. 2016 May 18;7:11641. doi: 10.1038/ncomms11641 (PMC4873973; doi:10.1038/ncomms11641)
Supplement: Supplementary Information — Supplementary Figures 1-19, Supplementary Tables 1-4, Supplementary Notes 1-4 and Supplementary References [file ncomms11641-s1.pdf]

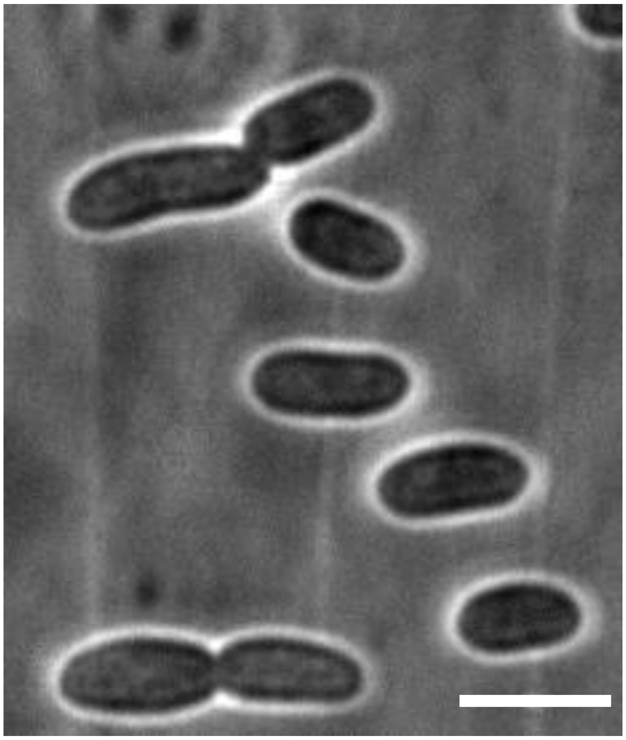

Supplementary Figure 1 | Phase contrast image of *E. coli* cells trapped using MACS. Scale bar (white) is 2 μm.

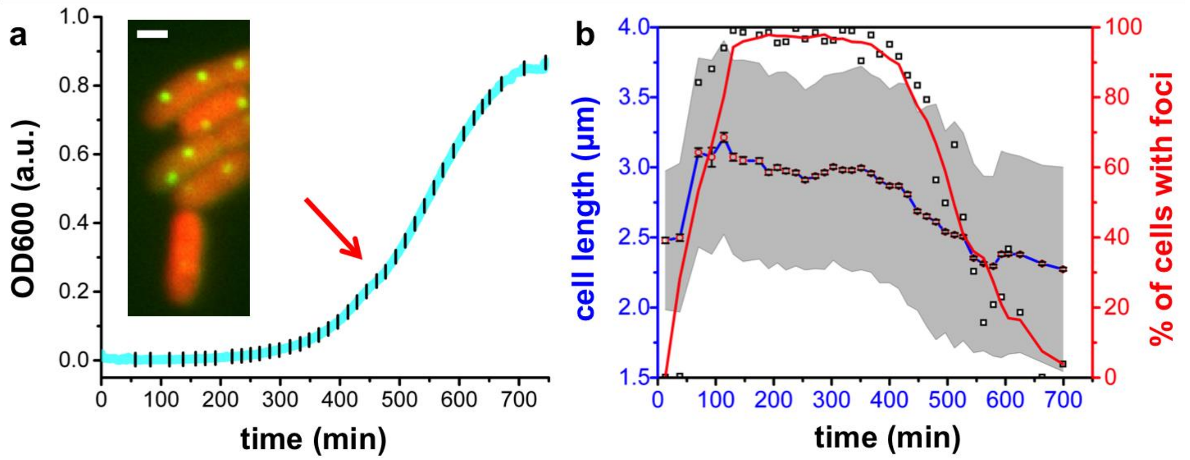

Supplementary Figure 2 | Microscopy imaging along the growth curve of *E. coli* via MACS **(a)** Cell density inside the growth chamber was monitored in real-time (cyan curve), and cells were imaged at time points specified by the black line-segments, each separated by ~15 min. A slight kink at ~450 min marked by a red arrow likely suggests a cumulative population-level change in the cell growth. Inset shows a representative snapshot of cells with a cytoplasmic marker (red) and the SeqA-mGFP foci (green), which marks the replication fork and serves as a proxy for DNA replication activity. Cell growth as well as the imaging was done within a temperature-controlled incubator at 37 ˚C. Cells were grown in M9 minimal media supplemented with 0.2% glucose, which resulted in a doubling time of 58 min in exponential phase. Scale bar (white) is 1 μm. **(b)** Cell length (shaded grey envelope, ± s.t.d.) and DNA replication activity over time. Reaching stationary after a rapid adjustment period, the cells collectively start dwarfing around t ~450 min, which corresponds to the kink in (a). Concomitantly, the DNA replication activity starts decreasing significantly at this point.

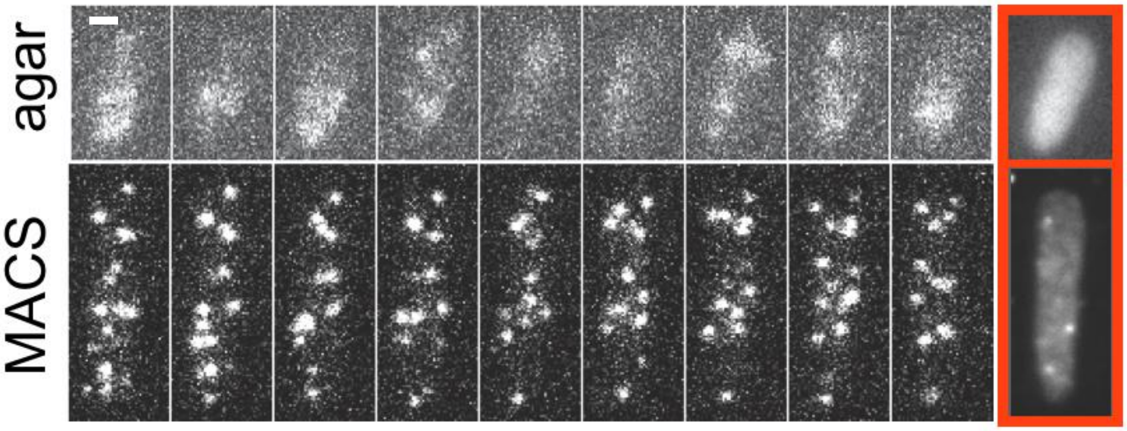

Supplementary Figure 3 | Nine consecutive frames from HILO imaging of *E. coli* cells expressing cytoplasmic mEos2 with a 30-msec exposure time: on agar pad (top), and on MACS (bottom) with P_{valve} = 20 psi. The fluorescence signal for cells imaged on agar appear smeared due to fast diffusion, whereas mEos2 molecules appear as discrete spots when cells were imaged using MACS. On the right of each panel, shown in the red boxes, are averages over the entire frames of the time-lapse movies (**Supplementary Movies 2 and 3**). Both images display diffuse signal due to averaging out. Scale bar (white) is 1 μm.

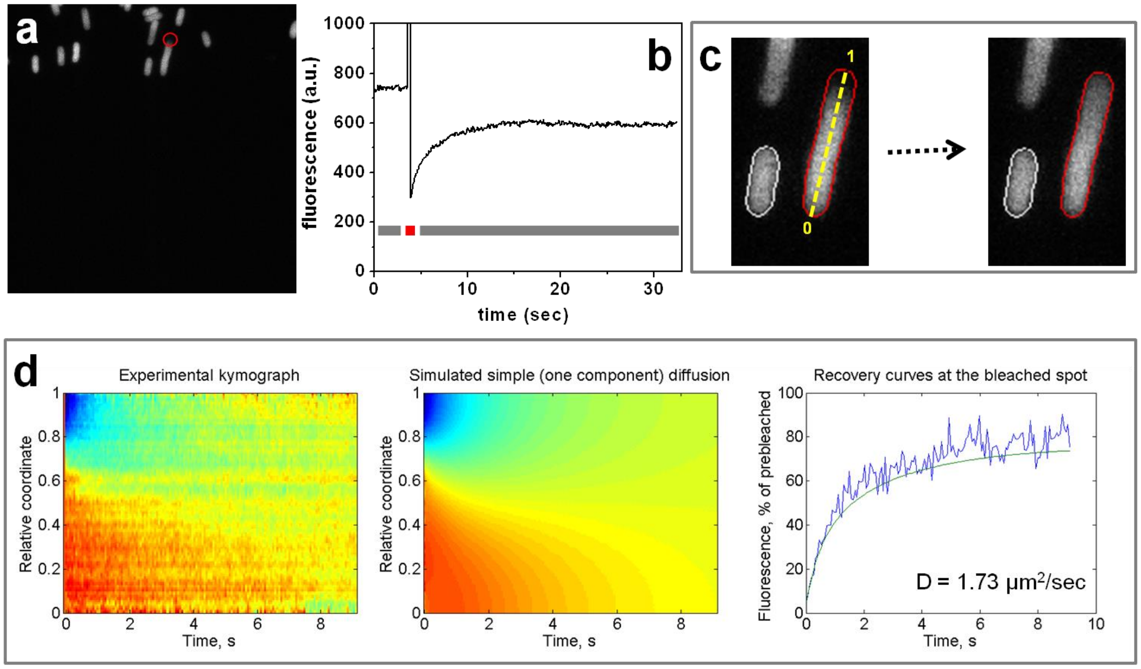

Supplementary Figure 4 | FRAP experiments and analysis. **(a)** *E. coli* cells trapped using MACS with P_{valve} = 5 psi. The region marked by the red circle was photo-bleached using a laser. **(b)** The fluorescence signal within the red circle over time showing pre-bleaching, bleaching and recovery periods. The red stripe marks the duration of the bleaching pulse while the grey stripes mark the periods of the pre-bleaching and recovery. **(c)** Diffusion coefficient analysis with MicrobeTracker as previously described[1]. The red line outlines the contour of the cell, which is shown immediately after its upper portion was photo-bleached (left). The intracellular signal was more homogenous after the signal recovery (right). **(d)** Measured and simulated kymographs for the fluorescence signal along the cell's long axis (marked by the yellow, dashed line in (c)), where blue represents low, and red represents high intensity. The fit to the recovery curve yields a diffusion coefficient of 1.73 μm^2 per sec.

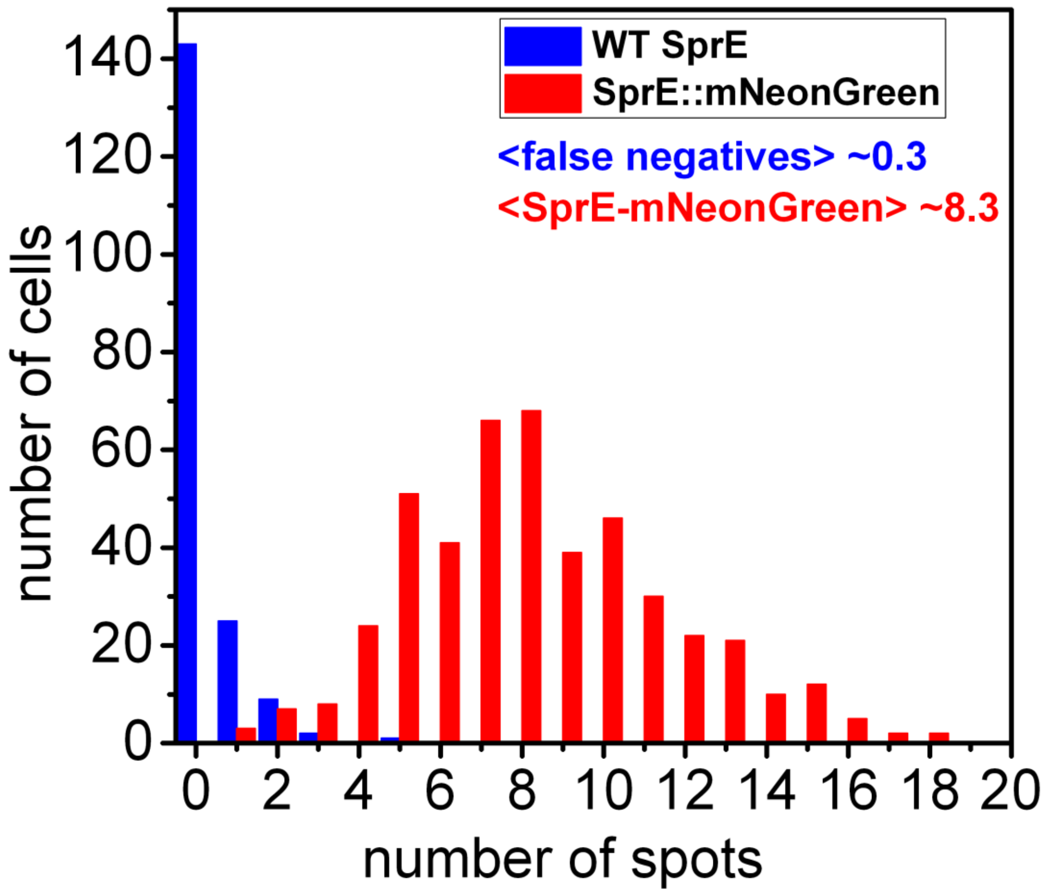

Supplementary Figure 5 | Distributions of the number of SprE spots per cell. A very low number of spurious spots (~0.3 per cell on average) are detected with the wild-type strain where SprE is untagged (WT SprE, blue histogram). Under otherwise identical conditions, the strain that expresses SprE tagged with mNeonGreen results in 8.3 spots per cell in average (SprE-mNeonGreen, red histogram). This analysis strongly suggests that the false positives due to spurious spots are minimal.

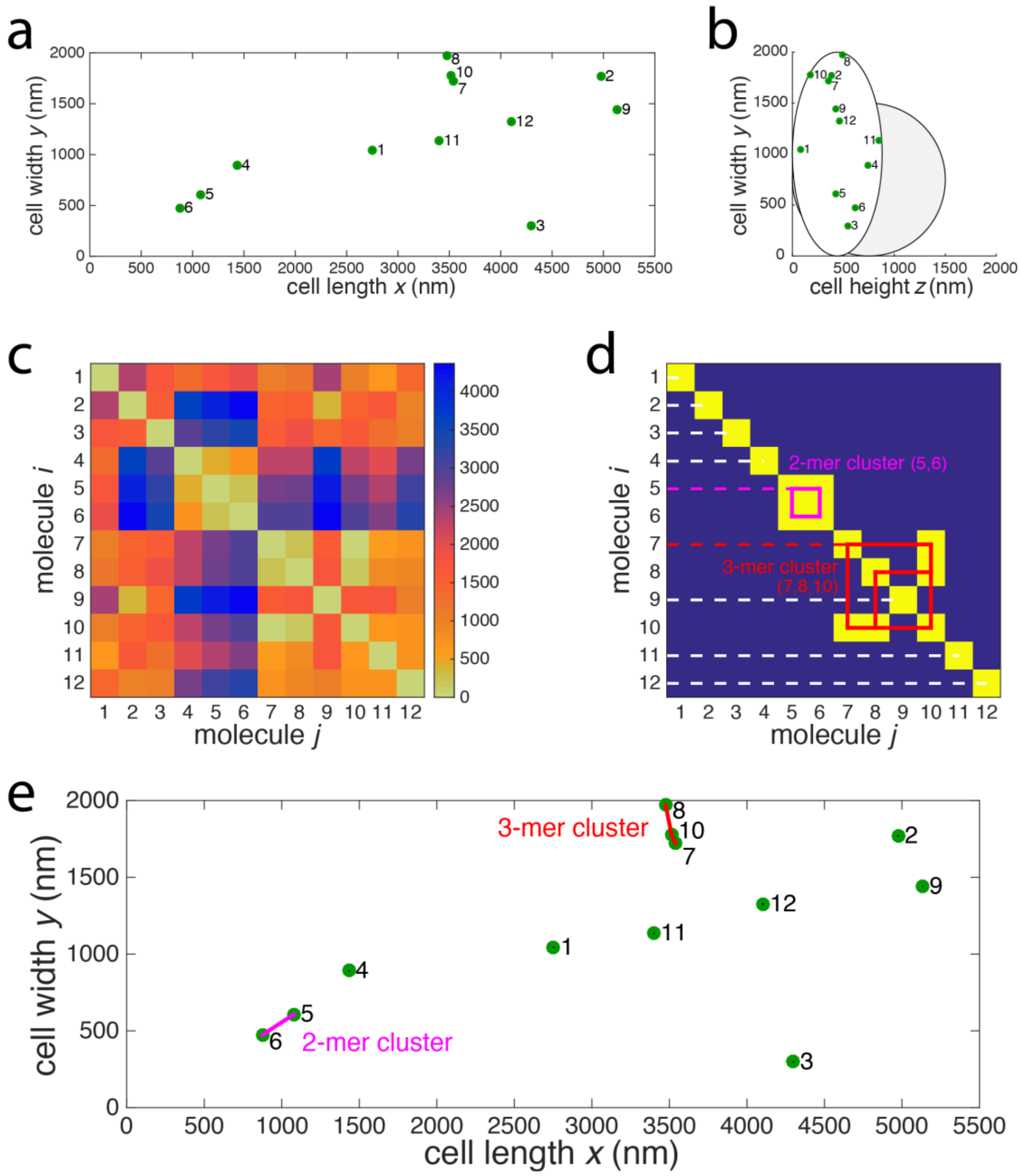

Supplementary Figure 6 | Numerical simulations for assessing the accuracy of single-molecule counting (**Supplementary Note 3**). A computer-generated plot shows the position of P = 12 molecules (green) in the longitudinal section (*xy*-plane) **(a)** and cross section (*zy*-plane) **(b)** of a virtual bacterial cell. Elliptical (white) or circular cross sections (gray) were used for the simulations. Physical pressing on the bacterial cell using MACS changes the cross section from circular to elliptical. **(c)** The pairwise Euclidian distances (in nm) in the longitudinal section (*xy*-plane) are calculated for all molecules and are recorded in the distance matrix. **(d)** Molecule pairs (*i*,*j*) that are less than 250 nm apart cannot be spatially resolved and are assigned a 1 (yellow) in the cluster matrix. Molecule pairs (*i*,*j*) that are more than 250 nm apart are assigned a 0 (blue). Using the cluster matrix, the algorithm loops over all rows, starting at row 1,

and determines whether the molecule of a given row is spatially resolved (dashed white line) or part of a cluster (magenta or red lines). Cluster assignment is performed using up to (P - 1) nested for loops to achieve maximum ("greedy") cluster growth. Rows that are not marked with a dashed line (e.g. row 6) where already assigned to clusters when the algorithm operated on a row with a smaller number (e.g. row 5 in the case of the molecule of row 6). **(e)** Plot of the longitudinal section (*xy*-plane) of the virtual bacterial cell with P = 12 molecules from above. Molecules that are less than 250 nm apart are connected by lines. Molecules 5 and 6 form a cluster (magenta lines) and molecules 7, 8, and 10 form a second cluster (red lines). The total number of observed molecules is 9. Three molecules were missed because of spatial clustering.

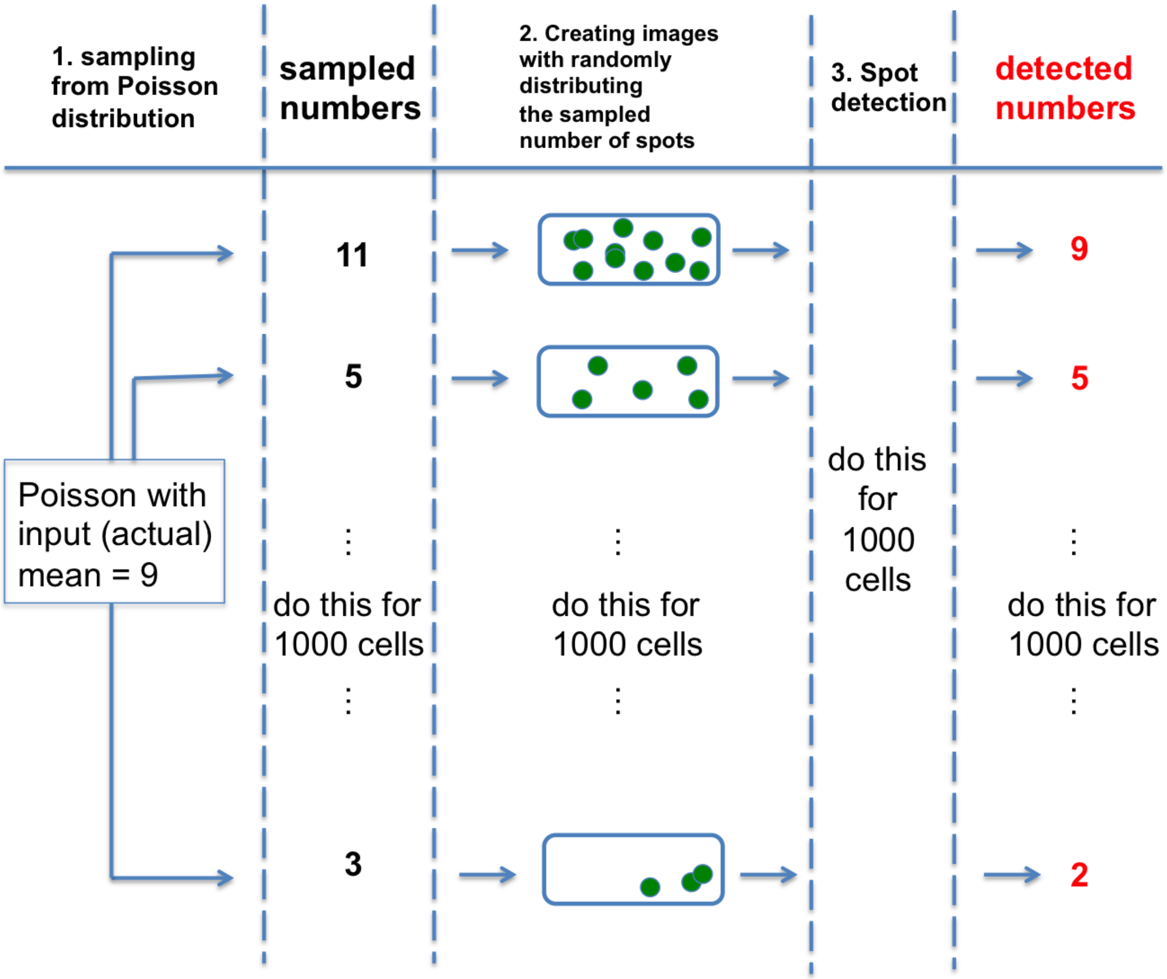

Supplementary Figure 7 | Schematic describing the algorithm for simulations carried out by generating images and analyzing the images using the spot-detection code.

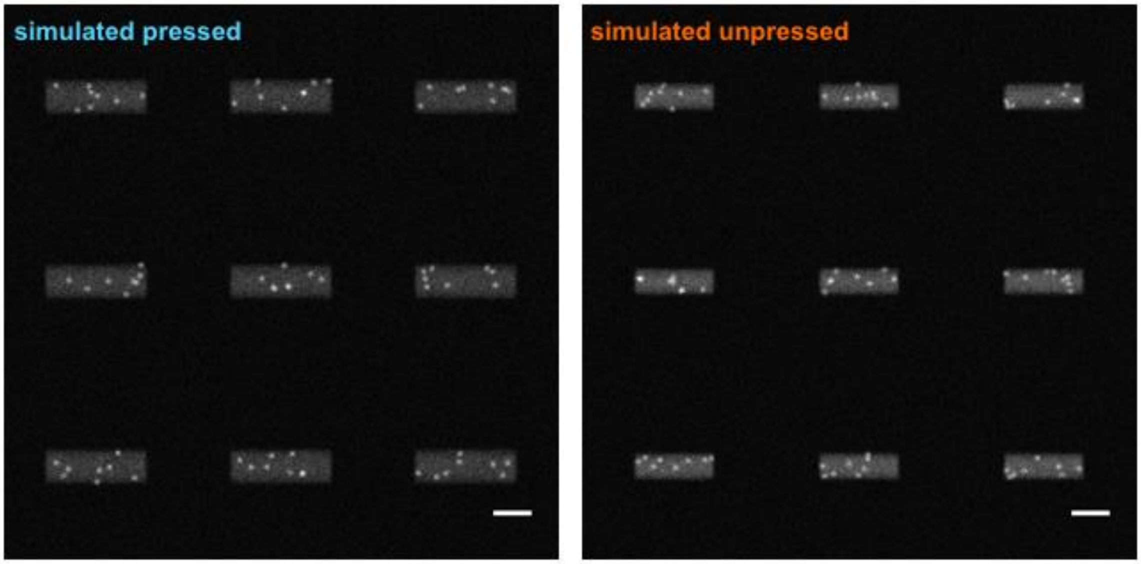

Supplementary Figure 8 | Simulated images with actual number of molecules = 8. Scale bar (white) is 2 μm.

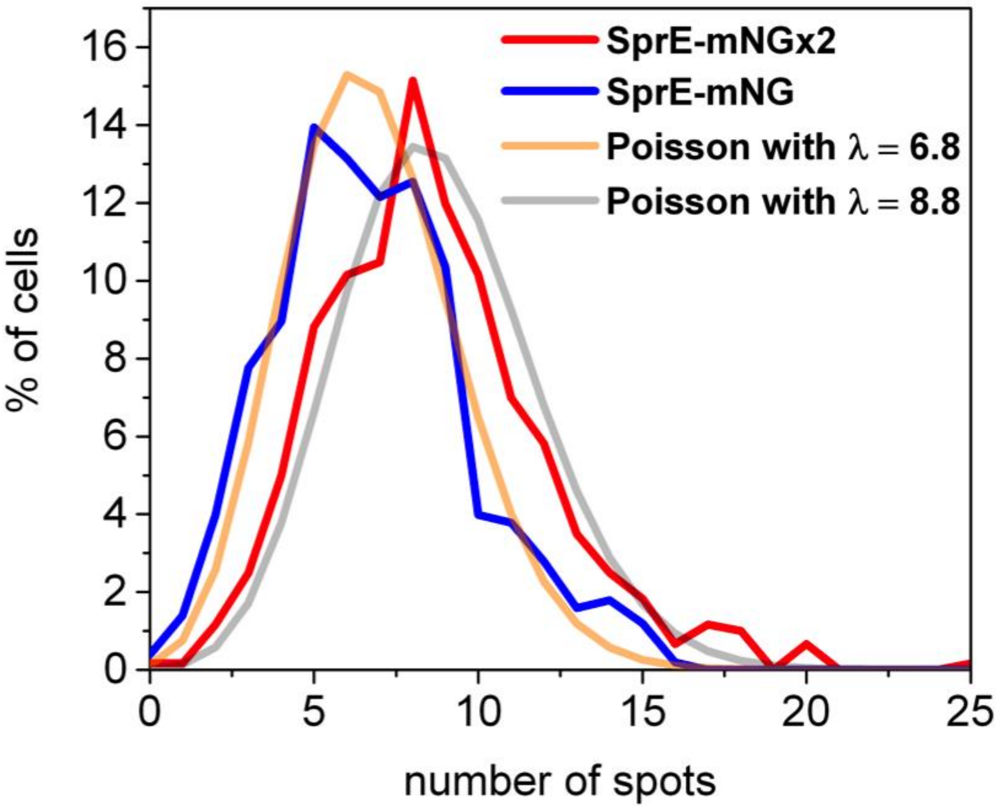

Supplementary Figure 9 | Distributions at $OD_{600} \sim 0.15$ using growth conditions as of Fig. 3d, for SprE tagged with a single mNeonGreen (SprE-mNG) vs. a tandem dimer (SprE-2xmNG). The measured distributions are shown overlaid with Poisson distributions of the same averages. SprE-2xmNG yields a ~30% higher count as expected due to the imperfect maturation yield of fluorescent proteins (the maturation efficiency of mNeonGreen appears to be approximately 80%). Both distributions shown here without size conditioning still closely follow Poisson.

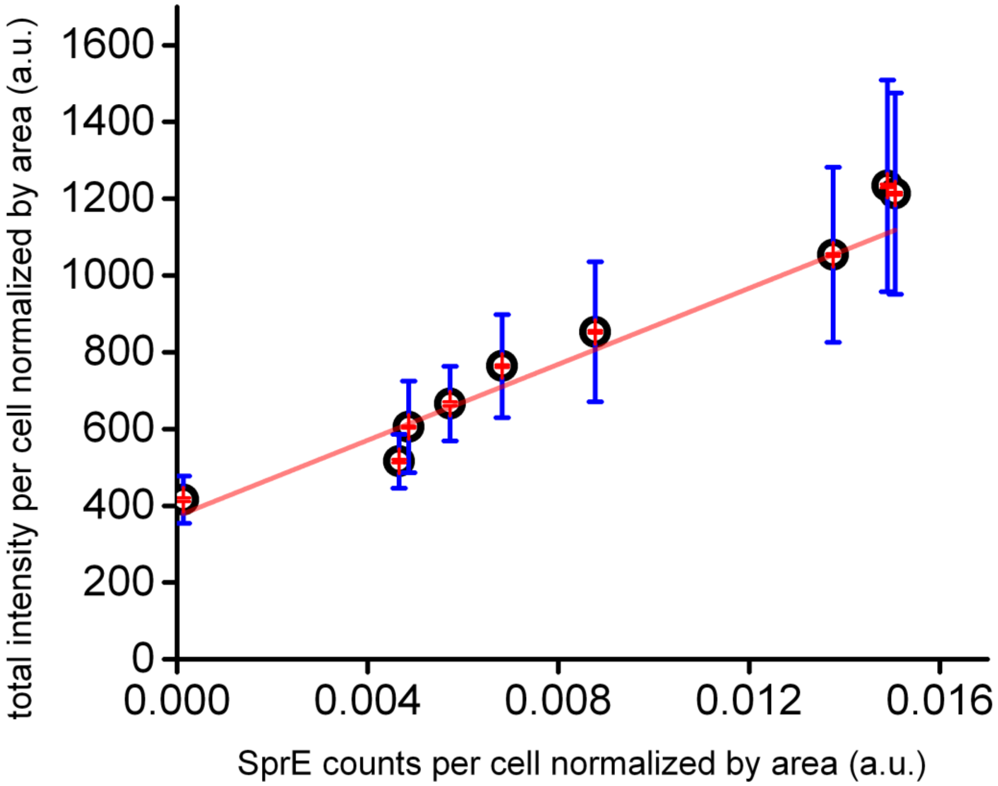

Supplementary Figure 10 | Linear dependence between total fluorescence intensity per cell and SprE concentration can allow for estimation of protein abundances at the population level for higher copies where spot overlap becomes a significant issue. The value for zero SprE copies was obtained from the WT background strain with the segmentation marker. Black circles are averages. Blue and red bars are standard deviation and standard error of the mean, respectively. The red line is a linear fit.

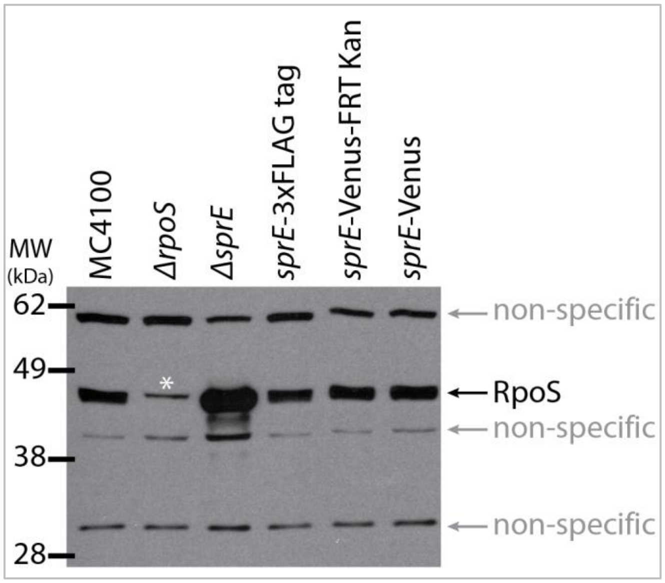

Supplementary Figure 11 | Western blot analysis shows that C-terminally tagged SprE fusion proteins behave identical to untagged, wild-type SprE and deliver the stress-response sigma factor RpoS to the protease ClpXP for degradation. RpoS accumulates significantly in the absence of SprE. Western blot samples were prepared from *E. coli* cells in late exponential phase (OD_{600} ~0.7). The monoclonal anti-RpoS antibody cross-reacts with several non-specific bands (indicated by gray arrows on the right), including a band at around 45 kDa (indicated by white asterisk) that runs only slightly above RpoS. The molecular weight of RpoS is 38 kDa but it runs at a higher apparent molecular weight slightly below 45 kDa. The RpoS protein is clearly detectable in the wild-type strain MC4100 (lane 1) but absent in the *ΔrpoS* strain (lane 2). The RpoS level is strongly increased in the *ΔsprE* deletion strain (lane 3) confirming SprE's essential role in controlling the RpoS protein level. The RpoS protein level is similar to the wild-type level in the SprE-3xFLAG tag (lane 4) and the SprE-Venus YFP strains (lane 5 & 6) showing that the behavior of untagged and tagged SprE is indistinguishable. SprE-Venus is derived from SprE-Venus-FRT Kan by eliminating the Kan resistance marker with pCP20.

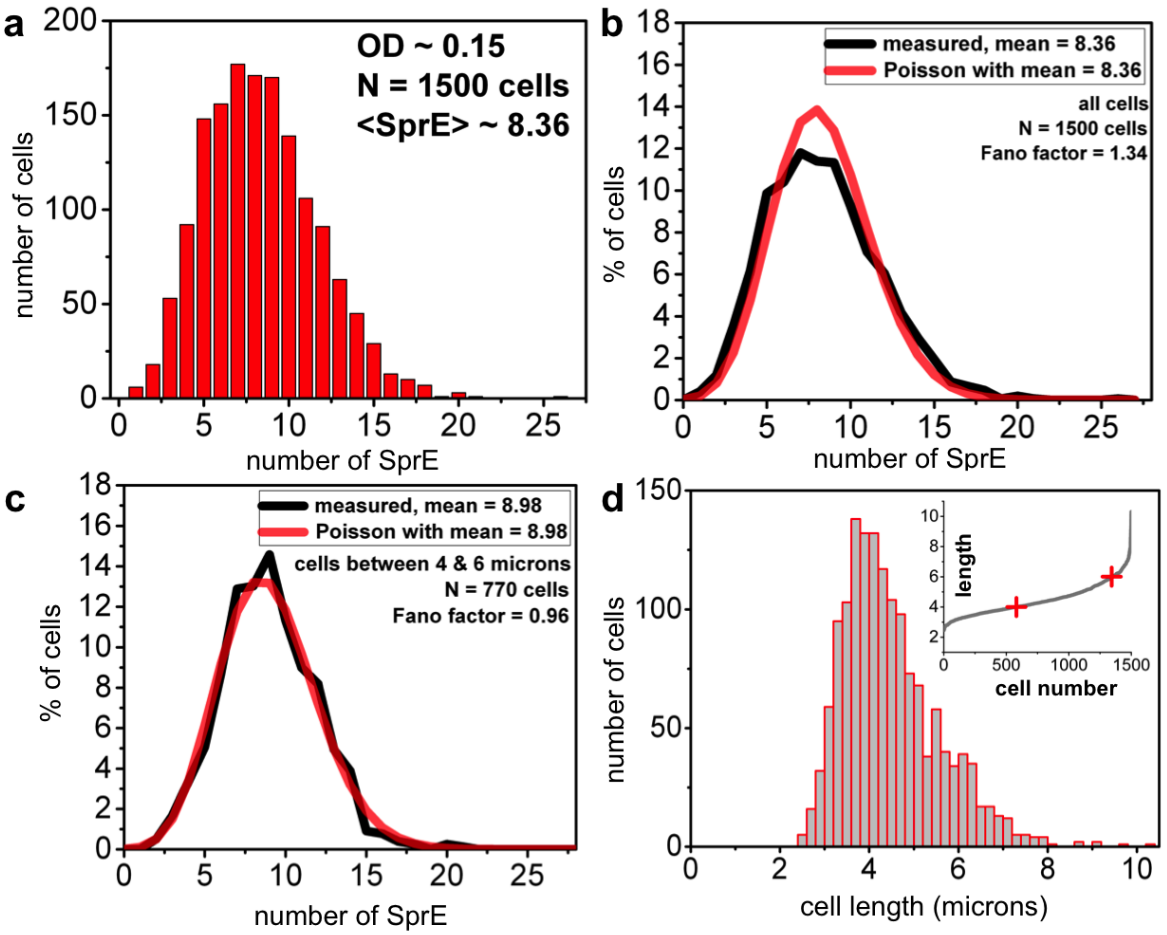

Supplementary Figure 12 | Effect of cell length conditioning on the number of SprE molecules per cell distribution and on the Fano factor. The dataset of OD_{600} = 0.15 is used. **(a)** SprE number distribution for all cells. **(b)** Distribution in (a) is normalized and overlaid with a Poisson distribution with the same average number of SprE molecules. A Fano factor of 1.34 suggests that the measured distribution is broader than Poisson when all cells are considered. **(c)** Normalized distribution considering only cells between 4 and 6 microns in length. This leads to higher average SprE numbers since longer cells tend to have more SprE molecules due to the fact that SprE numbers scale with cell size. In this case, the overlaid Poisson distribution closely resembles the measured distribution, which consistently exhibits a Fano factor of approximately 1. **(d)** Cell length distribution for all cells. Inset shows the range chosen (marked by red crosses) for the length conditioning in (c).

a

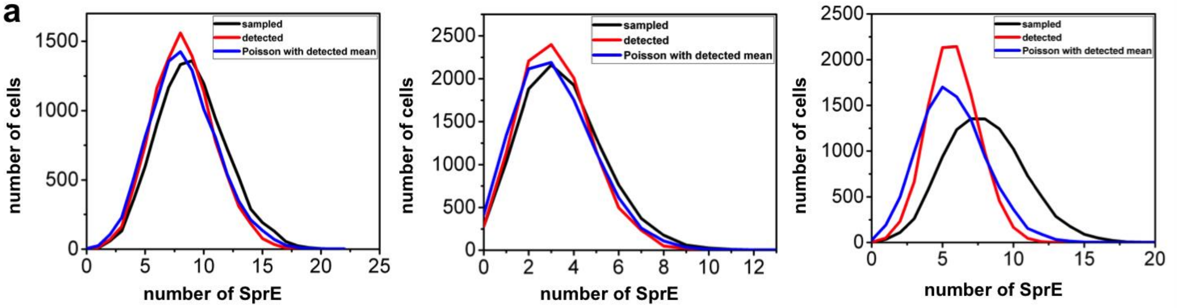

	mean (input)	mean (sampled)	mean (detected)	Fano factor (sampled)	Fano factor (detected)	cell length (nm)	cell width (nm)	zero-SprE (%)
OD600 = 0.04	9	8.98	8.3	0.99	0.84	5440	1830	0.04
OD600 = 1.2	3.5	3.51	3.2	0.99	0.82	2440	1470	2.8
OD600 = 1.88	8	8.00	5.7	1.00	0.57	1700	1350	0.03

b

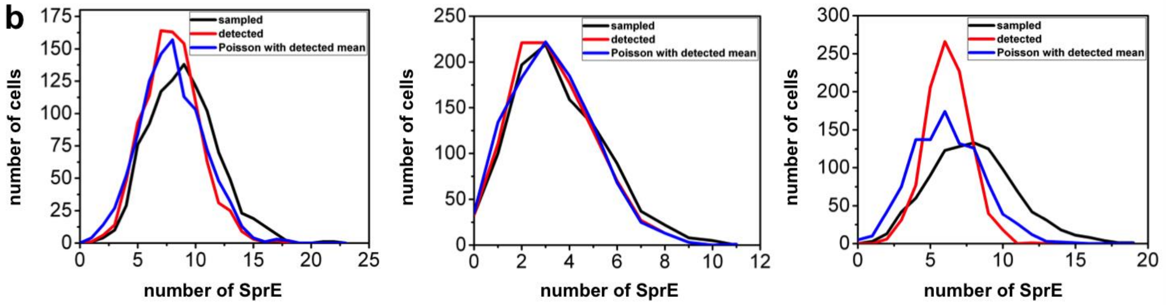

	mean (input)	mean (sampled)	mean (detected)	Fano factor (sampled)	Fano factor (detected)	cell length (nm)	cell width (nm)	zero-SprE (%)
OD600 = 0.04	9	8.96	7.9	0.96	0.73	5440	1830	0
OD600 = 1.2	3.5	3.53	3.3	1.06	0.93	2440	1470	3.5
OD600 = 1.88	8	7.96	6.2	1.12	0.37	1700	1350	0

Supplementary Figure 13 | Simulated distributions that are representative of various data points along the growth curve. Left, center and right panels correspond to OD_{600} = 0.04, t = 240 min; OD_{600} = 1.2, t = 495 min and OD_{600} = 1.88, t = 765 min, respectively. **(a)** Distributions are generated for 10,000 cells using numerical simulations (**Online Methods and Supplementary Fig. 6**). The black curves show the sampled Poisson distribution with the input mean number according to the experimentally detected average SprE count. The histograms for the detected number of spots according to the 250-nm resolution limit and the corresponding experimental cell geometries are shown as red curves. The blue curves represent a Poisson distribution with a mean of the detected numbers for comparison. The table summarizes the results of the simulations. **(b)** 1,000 images are generated for each condition using identical parameters to those of the numerical simulations (**Online Methods and Supplementary Fig. 7 and 8**). The simulated images were then analyzed with the spot-finding algorithm.

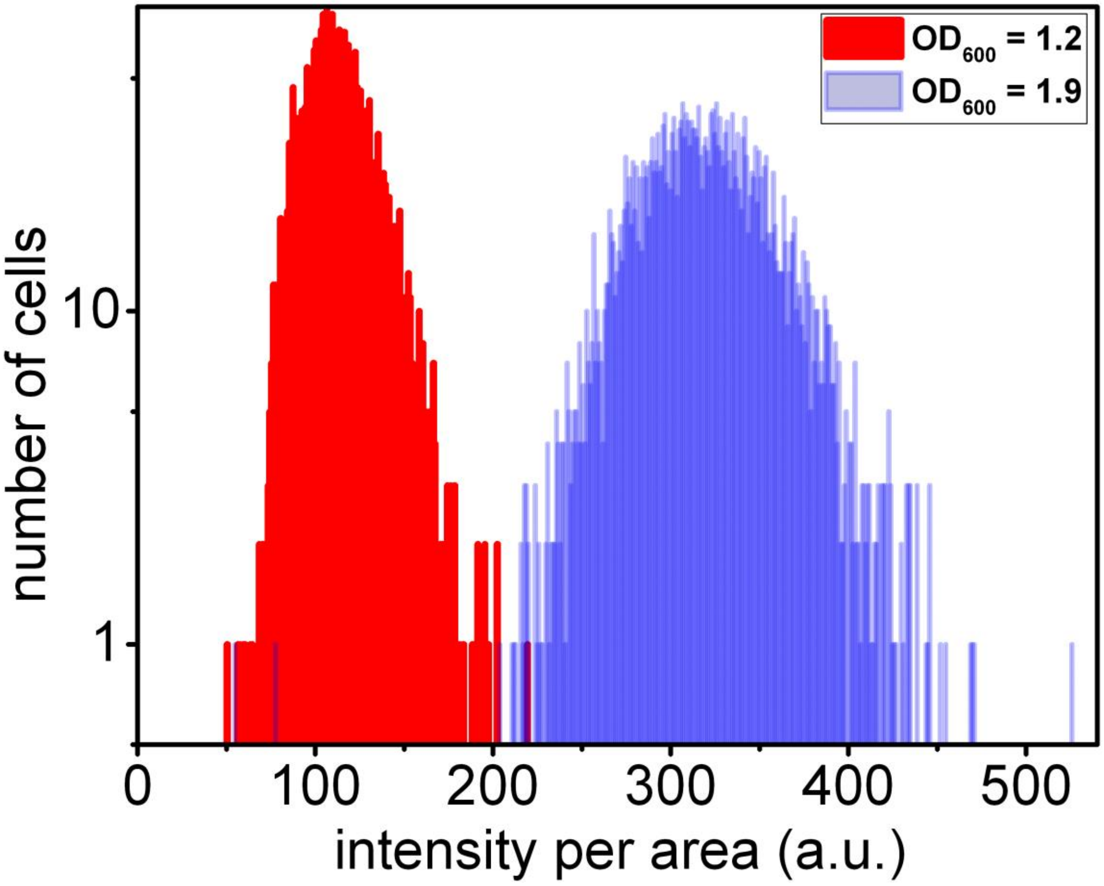

Supplementary Figure 14 | Histograms of the average fluorescence intensity of *E. coli* cells with the RpoS750-Venus at an OD_{600} of 1.2 (red) and an OD_{600} of 1.9 (blue), representing the SprE minimum and early stationary phase respectively. Essentially, no cell at OD_{600} of 1.2 exhibits a comparable intensity to that of the stationary-phase cells.

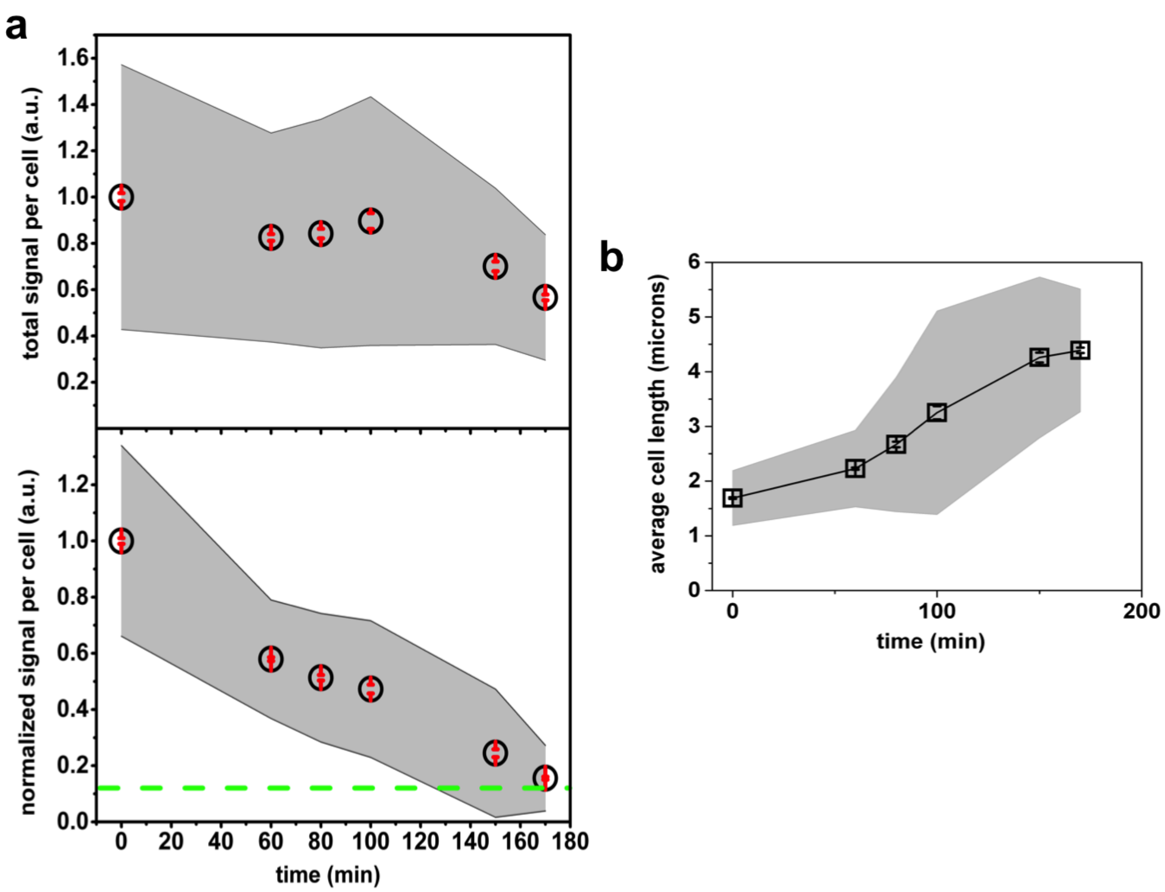

Supplementary Figure 15 | SprE dynamics upon exist from stationary phase. Cells were grown to deep-stationary phase for 5 days in M9 + 10% LB. The cells were then diluted into fresh media and monitored over time for **(a)** mNeonGreen signal and **(b)** cell length (open shapes and shaded grey area represent averages and standard deviation, respectively). Steadiness in total intensity for SprE-mNeonGreen until ~100 min suggests that the SprE is stable and is primarily diluted by cell division. By ~180 min the SprE levels return to that of the balanced-growth regime (marked by the green dashed line), also suggested by the SprE counts (data not shown). At this point, the average cell length is also representative of the balanced-growth.

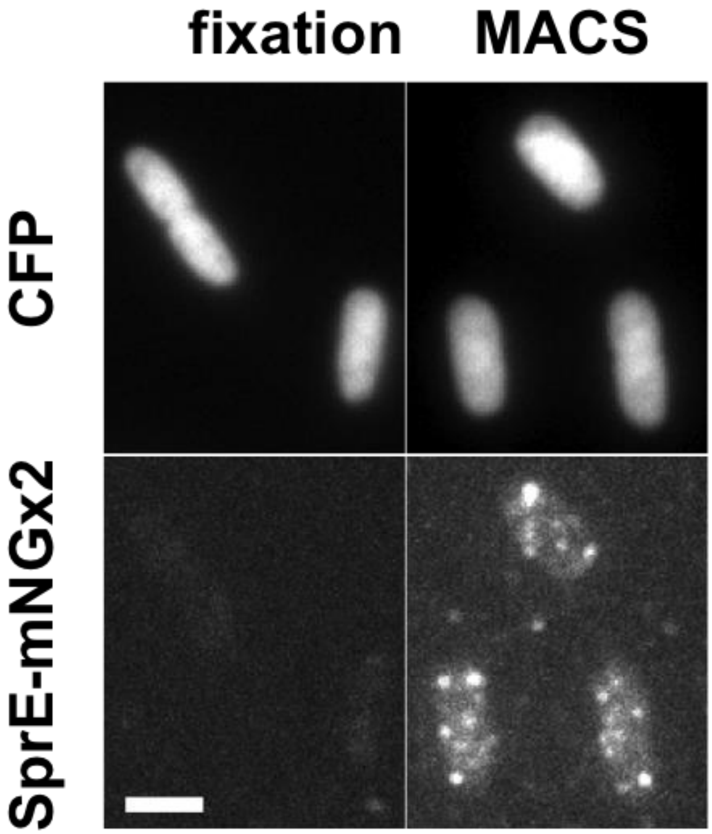

Supplementary Figure 16 | Comparison of chemical fixation (left) and MACS (right) for the same cell culture. Half the volume of a growing culture was used for fixation and the other half for MACS. Top and bottom panels display the CFP segmentation marker, and mNG tandem dimer tagged SprE, respectively. Chemical fixation results in significantly reduced number of observed spots even for the SprE tagged with tandem mNeonGreen. Cells shown here were fixed using paraformaldehyde, following the protocol described in Kuhlman et al.[2] Similar results were obtained using SprE-mNG as well as formaldehyde with various fixation times. Fixed cells were imaged on an agar pad, and imaging conditions were otherwise identical. Scale bar (white) is 2 μm.

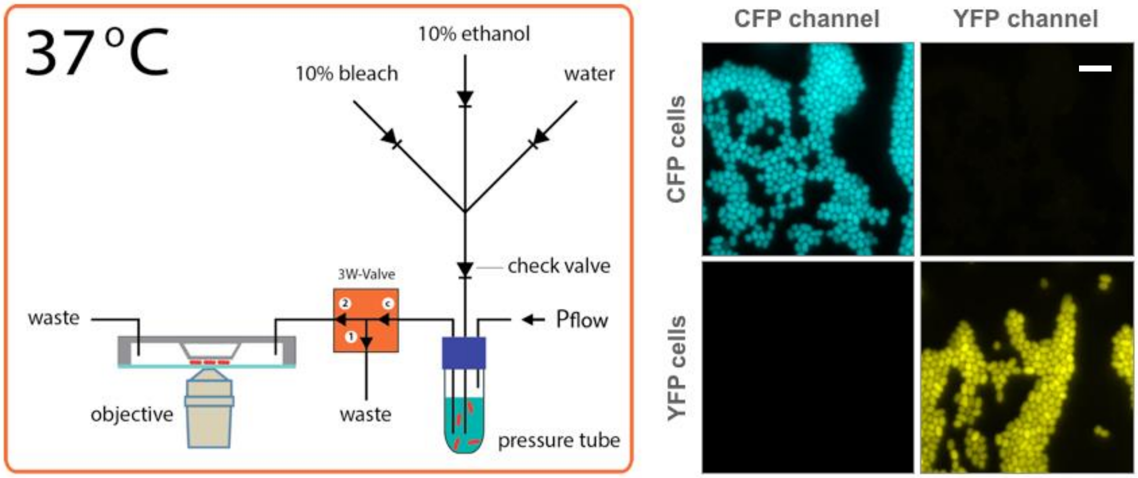

Supplementary Figure 17 | Detailed schematic of the automated imaging with MACS (left) and efficiency of the cleaning routine (right). **Left panel:** check valves ensure unidirectional flow. Three-way valve (3W-Valve) enable flow selection by diverting a common inlet (c) to two outlets (1 or 2). We directly send the sample from the pressure tube (PT) into MACS by setting the 3W-Valve to (2). To be able to rapidly empty the PT into the waste when rinsing or priming for the next sampling, we set the 3W-Valve to (1). The entire system is enclosed within a temperature-controlled incubator kept at 37 ˚C. **Right panel:** After each sampling, we run a cleaning routine by rinsing the PT and the MACS chip sequentially using 10% bleach, 10% ethanol, and water. Right panel shows the efficiency of the cleaning routine. When PT is initially filled with a CFP-expressing strain, cells are only detected in the CFP channel as expected. When we run the cleaning routine, and fill the PT with a YFP-expressing strain, cells are only detected in the YFP channel. A single representative snapshot is shown here but essentially no CFP-expressing cells were detected in >100 frames, which indicates that there is no carryover from the previous sampling of the YFP-expressing cells. Scale bar (white) is 5 μm.

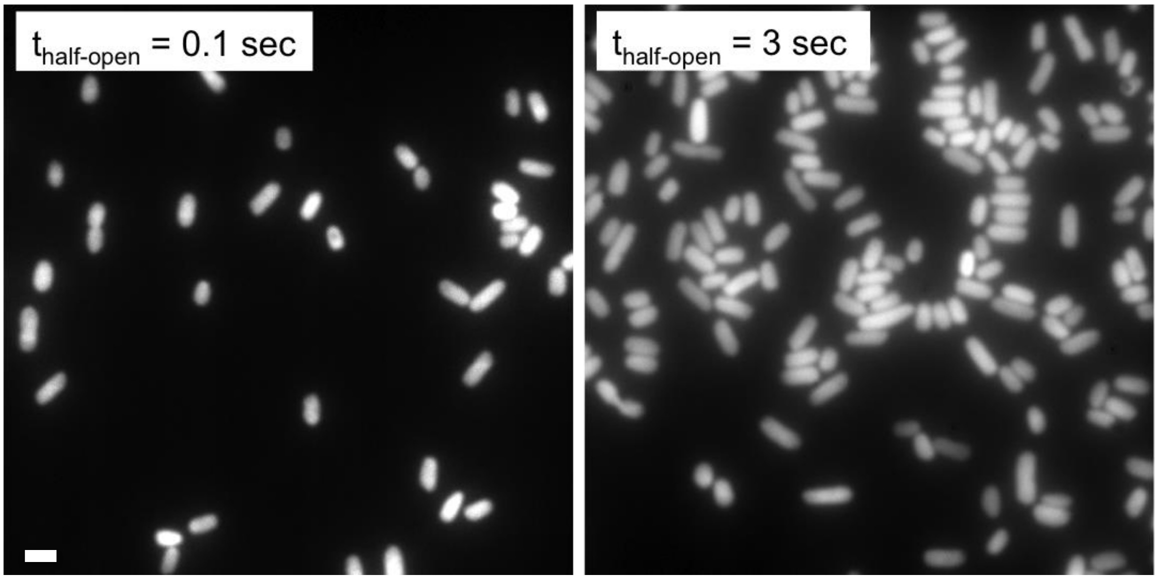

Supplementary Figure 18 | Effect of MACS parameters on cell density in the field of view. The cell density within the field of view can be adjusted by simply changing several parameters such as P_{flow} and P_{valve} as well as the duration of the half-open state. For instance, the two images correspond to two different $t_{half\text{-}open}$ values and other parameters being identical (t_{closed} = 1 sec, t_{open} = 1 sec, P_{flow} and P_{valve} are both 20 psi). This important feature is not only essential for single-molecule counting but may be useful when crosstalk of fluorescent intensities between neighboring cells, due to the size of the PSF, needs to be minimized for quantitative microscopy, since the fluorescence spillover from a bright cell would cause overestimation of the signal of an adjacent dimmer cell. Scale bar (white) is 2 μm.

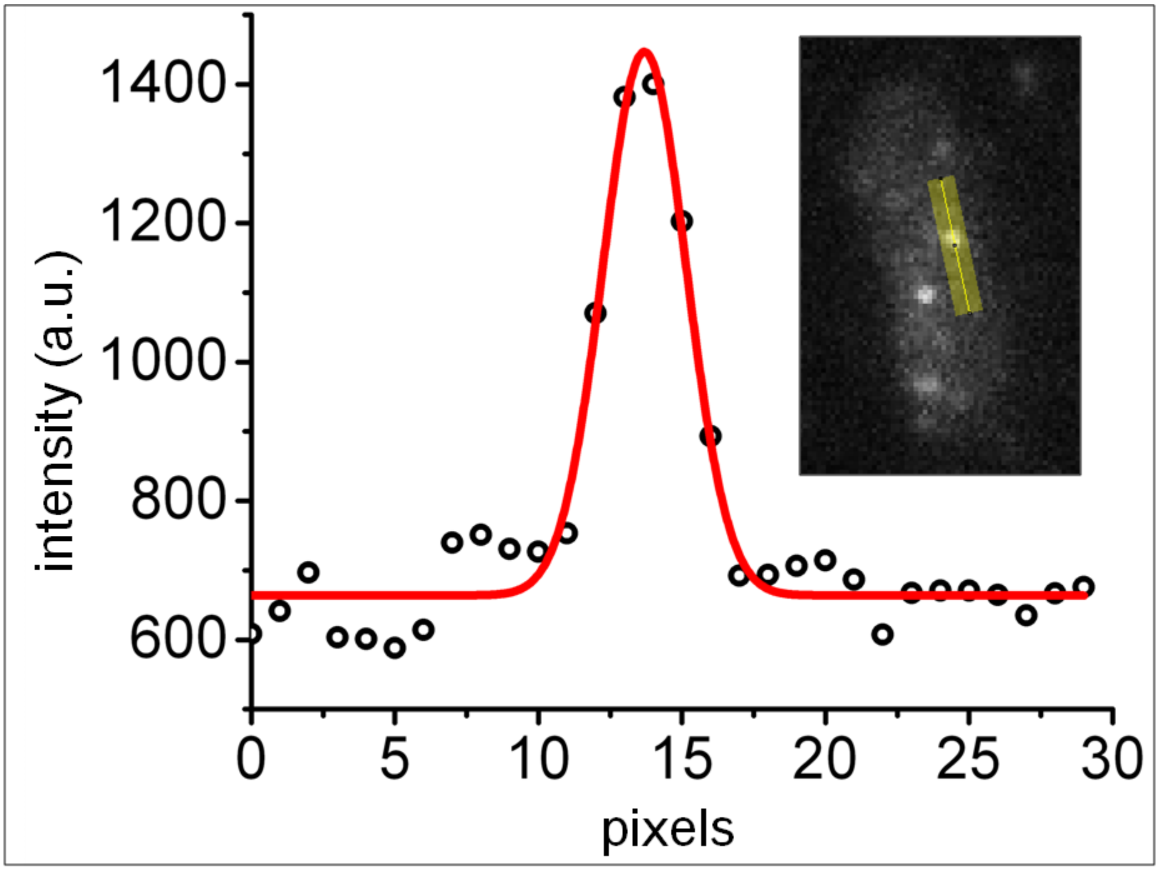

Supplementary Figure 19 | Determination of the point spread function of our optical system. Linear intensity profile (open circles) of a SprE-mNeonGreen molecule in an *E. coli* cell (inset) can be approximated with a 2D-Gaussian (red line) with a root-mean-squared size (σ) of 1.45 pixels (~93 nm for the magnification used on our system), which yields a resolution limit[3] of $r_{xy} = 2.4\sigma \sim 225$ nm.

Supplementary Table 1: List of parameters used for the simulations.

	**Fig. 2g**	**Fig. S10**	**Fig. S10**	**Fig. S10**
Number of cells, *N*	10,000	10,000	10,000	10,000
Mean number of molecules, $\langle P \rangle$	1 to 15 with step size = 1	9	3.5	8
Cell length (nm)	5,500	5,500	2,440	1,770
Cell width (nm)	2,000	2,000	1,450	1,330
Cell height (nm)	880	800	580	600
PDF_x	Uniform	Uniform	Uniform	Uniform
PDF_{zy}	Ellipse	Ellipse	Ellipse	Ellipse
Resolution in *x* and *y*, r_{xy} (nm)	250	250	250	250

Supplementary Table 2: *E. coli* strains used in this study.

Strain	Description	Antibiotic marker	Reference
MG1655	F^-, lambda$^-$, *rph-1*	-	Jensen[12]
MC4100	F^- *araD139 Δ(argF-lac)U169 rpsL150 relA1 flbB5301 deoC1 ptsF25 rbsR*	-	Casadaban[13]
JW5431	BW25113 *rpoS*::Kan	Kan	Baba[9]
BO37	MG1655 *glmS*::P_{RNAI}-mCherry$_{1-11}$-mKate-T1 terminator-FRT Kan FRT::*pstS*	Kan	This study
BO41	MG1655 *glmS*::P_{RNAI}-GFPmut2-T1 terminator-FRT Kan FRT::*pstS*	Kan	This study
BO56	MC4100 *galK*::P_{lac}-CFP-Amp *sprE*-2x mNeonGreen-AP tag-FRT Kan FRT	Amp, Kan	This study
DHL51	MC4100 *rpoS*::Tn10	Tet	This study
DHL193	MC4100 pKD46	Amp	Landgraf et al.[8]
DHL222	MC4100 *phoA*::P_{rpoS}-*rpoS750*-Venus-T1 terminator-FRT Kan FRT	Kan	This study
DHL241	MC4100 *sprE*::Tn10	Tet	This study
DHL307	MC4100 *sprE*-3xFLAG tag-FRT Kan FRT	Kan	This study
DHL331	MC4100 *sprE*-3xFLAG tag	-	This study
DHL339	MC4100 *rpoS*::Kan *sprE*-3xFLAG tag	Kan	This study
DHL394	DHL193 *sprE*-Venus-T1 terminator-FRT Kan FRT	Kan, Amp	This study
DHL399	MC4100 *sprE*-Venus-T1 terminator-FRT Kan FRT	Kan	This study
DHL749	MC4100 *ara*$^+$ pKD46	Amp	Landgraf et al.[8]
DHL812	MC4100 *sprE*-Venus YFP-T1 terminator	-	This study
DHL934	MC4100 pDHL917	Amp	This study
GL15	MC4100 *galK*::P_{lac}-CFP *phoA*::P_{rpoS}-*rpoS750*-Venus YFP-T1 terminator	Amp	This study
GL19	MC4100 *galK*::P_{lac}-CFP-Amp	Amp	This study
GL45	MC4100 *galK*::P_{lac}-CFP-Amp *sprE*-mNeonGreen	Amp	This study
GL64	MC4100 *galK*::P_{lac}-CFP-Amp *rpoS*::Kan *sprE*-mNeonGreen	Amp, Kan	This study
Wy	MC4100 *galK*::P_{lac}-YFP-Amp	Amp	Hegreness et al.[14]
Wc	MC4100 *galK*::P_{lac}-CFP-Amp	Amp	Hegreness et al.[14]

Supplementary Table 3: Plasmids used in this study.

Plasmid	Description	Antibiotic marker	Reference
pCP20	Expression plasmid with the yeast Flp recombinase gene	Amp, Cm	Datsenko & Wanner[15]
pDHL16	pUC19-P_{rpoS}-*rpoS750*	Amp	This study
pDHL17	pUC19-Venus-T1 terminator	Amp	This study
pDHL19	pUC19-FRT Kan FRT	Amp, Kan	Landgraf et al.[8]
pDHL23	pUC19-P_{rpoS}-*rpoS750*-Venus-T1 terminator	Amp	This study
pDHL39	pUC19-P_{rpoS}-*rpoS750*-YFP-T1 terminator-FRT Kan FRT	Amp, Kan	This study
pDHL146	pUC19-Venus YFP-T1 terminator-FRT Kan FRT	Amp, Kan	Landgraf et al.[8]
pDHL229	pUC19-3xFLAG tag-FRT Kan FRT	Amp, Kan	This study
pDHL580	pUC19-linker-mGFPmut3-FRT Kan FRT	Amp, Kan	Landgraf et al.[8]
pDHL583	pUC19-linker-Dronpa-FRT Kan FRT	Amp, Kan	Landgraf et al.[8]
pDHL655	pUC19-linker-mGFPmut3-AP tag-FRT Kan FRT	Amp, Kan	This study
pDHL694	pUC19-linker-Dronpa-AP tag-FRT Kan FRT	Amp, Kan	This study
pDHL830	pSC101-P_{LlacO1}-Zif268-linker-AP tag-Amp	Amp	This study
pDHL844	pUC19-linker-mEos2-FRT Kan FRT	Amp, Kan	Landgraf et al.[8]
pDHL917	pSC101-P_{LlacO1}-mEos2-linker-AP tag-Amp	Amp	This study
pDML22	pUC19-linker-mNeonGreen-FRT Kan FRT	Amp, Kan	This study
pDML199	pUC19-linker (SGGGG)-mNeonGreen-linker (5xGA)-mNeonGreen-AP tag-FRT Kan FRT	Amp, Kan	This study
pKD13	Template plasmid for gene disruption. The Kan gene is flanked by FRT sites.	Kan	Datsenko & Wanner[15]
pKD46	λRed recombinase plasmid	Amp	Datsenko & Wanner[15]
pPM16	pSC101-P_{LlacO1}-Venus-T1 terminator	Amp	Landgraf et al.[8]
pUC19	High-copy-number cloning vector	Amp	Invitrogen

Supplementary Table 4: Primers used in this study.

Primer name	DNA sequence
DHL_P13_F	ccttgaattcaacgtgaggaaatacctggatttttcc
DHL_P14_R	aagggagctccgtggtatcttccggaccgttc
DHL_P15_F	ccttgagctcagtaaaggagaagaacttttcactggagttg
DHL_P16_R	aaggcccgggggcggatttgtcctactcaggag
DHL_P21_F	ccttgagctcagcggtggcggtggcagtaaaggagaagaacttttcactggagttg
DHL_P46_R	agaacagcctgccagccatagc
DHL_P47_F	ttgcgttttcccttgtccagatag
DHL_P79_R	Cggtgccctgaatgaactgc
DHL_P80_F	gcccagtcatagccgaatagcc
DHL_P81_R	gcgacgatagtaccaccagcc
DHL_P93_F	aagaagttattgaagcatcctcgtcagtaaaaagttaatcttttcaacagcaacgtgaggaaatacctggatttttcc
DHL_P94_R	cagcaaaaaaaccacccggcagcgaaaattcactgccgggcgcggttttaattccggggatccgtcgacc
DHL_P101_F	tcgaaagaactgtgtgcgcagg
DHL_P104_R	aaagttctctcggcagcgcc
DHL_P105_F	ccttgagctcgactacaaagaccatgacggtgattataaag
DHL_P106_R	aaggcccgggccatatgaatatcctccttagttcctattcc
DHL_P107_F	gccaaatatggggaaccggtggtcgactgcgcttgatgttgtctgcagaagactacaaagaccatgacggtgattataaag
DHL_P108_R	agccgacattagcaggtaatgcaaatttagcccgcgttatcgtttgctcaccatatgaatatcctccttagttcctattcc
DHL_P120_F	ttaattatcgtcaattggttgccgc
DHL_P121_R	ggggatcttgaagttcctattccg
DHL_P168_F	gccaaatatggggaaccggtggtcgactgcgcttgatgttgtctgcagaaagcggtggcggtggcagtaa
DHL_P169_R	agccgacattagcaggtaatgcaaatttagcccgcgttatcgtttgctcaattccggggatccgtcgacc
DHL_P255_F	ccttgagctcagcggtggcggtggcagtaacgtgattaaaccagacatgaagatcaagc
DHL_P315_R	aaggcccgggtttgtatagttcatccatgccatgtgtaatc
DHL_P316_F	ccggggagtcctccggcggcggcctgaacgacatcttcgaggcccagaagatcgagtggcacgagtaag
DHL_P317_R	ccggcttactcgtgccactcgatcttctgggcctcgaagatgtcgttcaggccgccgccggaggactcc
DHL_P348_R	aaggcccgggcttggcctgcctcggcagc
DHL_P397_F	agcttctgagtcctccggcggcggcctgaacgacatcttcgaggcccagaagatcgagtggcacgagtaataagctgagt
DHL_P398_R	ctagactcagcttattactcgtgccactcgatcttctgggcctcgaagatgtcgttcaggccgccgccggaggactcaga
DHL_P485_F	accgaattcattaaagaggagaaaggatccatgagtgcgattaagccagacatgaagatc
DHL_P486_R	agatgtcgttcaggccgccgccggaggactcagaagctcgtctggcattgtcaggcaatc

DML_P118_F	gccagtgaattcgagctcagcggtggcggtggcagtaaaggcgaggaggataacatggcc
DML_P119_R	ataggaacttcgaagcagctccagcctacaccccgggttacttgtacagctcgtc
DML_P635_F	gatgggcatggacgagctgtacaagggagcaggtgctggtgctgg
DML_P636_R	tcgttcaggccgccgccggaggactccccgggcttgtacaattcgtccatacccataacg
DML_P637_F	cgttgtaaaacgacggccagtgaattcgagctcagcggtggcggtggcagtaacggcgag gaggataacatggcc
DML_P638_R	gcGccagcaccagcacctgctcccttgtacagctcgtccatgccc
DML_P674_F1	tgggcgcgttattgaagcagg
DML_P675_R1	ttactgccaccgccaccgctttctgcagacaacatcaagcgcag
DML_P676_F2	ggtcgacggatccccggaatgcaaacgataacgcgggctaaatt
DML_P677_R2	gcagcatctctttcgggatggc
DML_P682_R	tattgaattaatggcttatcgacaagtgg

Supplementary Note 1: The simulations were performed with variables representing the confinement volume (i.e. cell geometry), diffusion coefficient of molecules, photophysical properties of the fluorescent tags, diffraction-limited imaging and EMCCD specifications. For simulating single-molecule images acquired on agar pads, the confinement volume was represented as a cylinder that has a circular cross section with a diameter equal to the average diameter of an *E. coli* cell. This diameter was measured using the fluorescence images of the cells expressing cytoplasmic CFP as a cell marker. In the case of MACS, the pressing causes cell flattening, which we assume results in cells with an elliptical cross-section. Therefore, single-molecule images acquired with MACS were simulated with a confinement volume modeled as a cylinder with an elliptical cross section. The major axis of the elliptical cross section was calculated from images of cells expressing cytoplasmic CFP. The short axis was estimated using the assumption that the circumference of the cell did not change from the circular to elliptical transformation due to pressing, which typically corresponds to an estimation of ~50% decrease in cell height. Diffusive traces of single molecules were created using a random walk algorithm[4] with the molecular diffusion constant estimated from FRAP measurements (D ~0.1 μm^2 per s). The diffuser emits photons along the entire trajectory. The number of photons per emitter was sampled from a Poisson distribution with a mean of 1000 photons per molecule. The emitted photons fall on top of the EMCCD pixels with a spread given by the point-spread function of the diffraction-limited imaging optics. The point-spread function is approximated as a two-dimensional symmetric Gaussian with $\sigma = 0.22\ \lambda/NA$ (~80 nm for light with a wavelength of λ ~520 nm and an objective lens with a numerical aperture of NA = 1.45) on our setup[3]. The detection noise is modeled using a Poisson distribution. The photons were converted to electrons according to the quantum efficiency and the EM gain of the EMCCD camera. The electrons are converted to counts according to the specified inverse system gain of the EMCCD. Finally, background noise originating from dark current and cellular autofluorescence was added to each pixel. Dark current, background noise and gain noise parameters were estimated from the EMCCD specifications. Cellular autofluorescence signal counts were estimated from control cells with wild-type (untagged) SprE. Simulated images were then analyzed according to the abovementioned spot-finding algorithm.

Supplementary Note 2: A previously published theoretical model predicts that the cells are critically crowded, i.e. the protein densities that are optimal for the reaction rates happen to be close to the actual protein densities in the cells[5]. Simply put, the cytoplasm may have just enough water to support diffusion[6] perhaps in order to ensure high effective concentrations and thereby higher rates of bimolecular reactions, and eliminating some water, ever-slightly, causes cells to transition into a regime where macromolecules barely diffuse at all. This prediction is consistent with our experimental observation of the single-molecule visualization when we use MACS to press on the cells and slow down the diffusion of the cytoplasmic proteins.

Supplementary Note 3: In conventional fluorescence microscopy, an individual point source like a single mNeonGreen molecule emits light at ~517 nm[7], which is spread by diffraction, resulting in an intensity distribution on the detector of the camera chip. This intensity distribution is known as the point-spread function (PSF) and has a width that is over two orders of magnitude larger than the nanometer-sized fluorescent protein. Molecules that are in close spatial proximity cannot be individual resolved if their PSFs overlap substantially, which would result in protein undercounting. This resolution limit, based on the Rayleigh criterion, is defined as $r_{xy} = \frac{0.61 \times \lambda_{em}}{NA}$ where λ_{em} is the emission wavelength of the fluorophore and NA is the numerical aperture of the microscope objective. For mNeonGreen and a 100× objective (NA 1.45), r_{xy} is about 217 nm, which is in good agreement with the PFS measurement that we performed on our microscope setup (**Supplementary Fig. 19**). As a conservative estimate, we used $r_{xy} = 250$ nm for the computer simulations.

In total, P molecules are drawn from a Poisson distribution with mean $\lambda = \langle P \rangle$ and placed into a three-dimensional cell with fixed cell length, cell width, and cell height (**Supplementary Table 1** and **Supplementary Fig. 6 a and b**). The exact x, y, and z position of each individual molecule is determined by drawing three random numbers (x, y, z) from continuous uniform distributions. For the molecule position along the cell length coordinate, x is drawn from a continuous uniform distribution with minimum and maximum values of x_a and x_b (e.g. 0 and 5,500 nm). For the position along the cell width and cell height coordinates, y and z are drawn form continuous uniform distributions with minimum and maximum values of y_a and y_b (e.g. 0 and 2,000 nm) and of z_a and z_b (e.g. 0 and 880 nm), respectively. The algorithm then tests whether the molecule falls within an ellipse with a semi-major axis of $cell\ width/2$ (e.g. 1,000 nm) and a semi-minor axis of $cell\ height/2$ (e.g. 440 nm) (**Supplementary Fig. 6b**). The condition C for this test is $C = \frac{(y - y_{center})^2}{(cell\ width/2)^2} + \frac{(z - z_{center})^2}{(cell\ height/2)^2} \leq 1$ and new random numbers are drawn for x, y, and z if $C > 1$ (i.e. the molecule falls outside the cell volume if $C > 1$). If the cross section of the cell is a circle instead of an ellipse, *cell width* equals *cell height* and C can be simplified to $C = \frac{(y - y_{center})^2 + (z - z_{center})^2}{(cell\ width/2)^2} \leq 1$.

The height of an average *E. coli* cell is larger than the depth of field (DOF) of a typical high-resolution fluorescence microscope, including our setup. Hence not all molecules are captured within a single focal plane, which could result in protein undercounting. When we press on the cells using MACS, the cell height is substantially reduced and all fluorescent molecules appear within a single focal plane. We hence limit the spatial overlap analysis to two dimensions (i.e. the cell length x *vs.* cell width y plane). The algorithm calculates for all molecules in a given cell the pair-wise Euclidean distances between the molecules using the following formula: $r_{ij} = \sqrt{\left(x_i - x_j\right)^2 + (y_i - y_j)^2}$ (**Supplementary Fig. 6c**). This distance matrix is converted into a logical matrix, which we call the cluster matrix (**Supplementary Fig. 6d**). Pairs of molecules that are less than r_{xy} apart from each other are assigned the value 1 (i.e. spatial overlap) whereas molecules that are more than r_{xy} apart are assigned a 0 (i.e. spatially resolved). Every row of the distance matrix is then assigned a flag that represent the

'status' of the molecule with values of either 1 (i.e. active) or 0 (i.e. inactive). After placement of the molecules, all rows have a status of 1 (active). The algorithm then starts with row i = 1, which corresponds to molecule i = 1, and searches for first-degree nearby neighbors (i.e. molecules that are less than r_{xy} away from molecule i = 1). If molecule j is a first-degree nearby neighbor of molecule i = 1, then the corresponding i,j-entry in the cluster matrix would be 1 (see next paragraph). If no first-degree nearby neighbors of molecule i were found, the 'status' flag of molecule i is set to 0 (inactive) and the code then proceeds with the next row (e.g. i = 2). Rows that have a status of 0 (inactive) are skipped by the algorithm (e.g. row 6; see below). This is necessary to prevent assigning and counting of the same molecule multiple times.

When the algorithm encounters a row i, e.g. i = 5, that has a first-degree nearby neighbor j, e.g. j = 6, with an i,j-entry of 1 in the cluster matrix (**Supplementary Fig. 6d**), the algorithm will also search for potential higher-degree nearby neighbor(s) and neighbors of neighbors to connect elongated molecule clusters. The 'status' flag of molecules that are assigned to a cluster is set to 0 (inactive). Using nested for loops, the algorithm checks for up to the (P-1)th-degree nearby neighbors (e.g. if P = 10, the code searches for the first- and potentially up to the ninth-degree nearby neighbors) given that a lower-degree nearby neighbor exists. This strategy assures that all connected molecules are found and are correctly assigned to the respective clusters. For example, molecule i = 7 has a first-degree nearby neighbor (i.e. molecule 10) and a second-degree nearby neighbor (i.e. molecule 8, which is indirectly linked to 7 via 10). Finally the number of clusters is determined and added to the number of molecules that are not part of a cluster to determine the total number of detected molecules. For the example from above (**Supplementary Fig. 6a,b**), the cell with P = 12 molecules has 7 spatially resolved molecules and two clusters with 2 and 3 molecules, respectively (**Supplementary Fig. 6e**). The total number of detected molecules is 9. Three molecules were missed because of the width of the PFS and the resulting spatial clustering.

Supplementary Note 4: Construction of *E. coli* strains, plasmids and primers used in this study.

E. coli strain construction

All *E. coli* strains that were used in this study are listed in **Supplementary Table 2**.

Strain BO37 was built by P1 transducing the *glmS*::P_{RNAI}-mCherry$_{1\text{-}11}$-mKate-T1 terminator-FRT Kan FRT::*pstS* allele into MG1655. The P1 lysate was a gift from Nate Lord (Paulsson Lab, Harvard Medical School). The P1 transduction was performed according to the protocol from the Sauer lab (see http://openwetware.org/wiki/Sauer:P1vir_phage_transduction).

Strain BO41 was built by P1 transducing the *glmS*::P_{RNAI}-GFPmut2-T1 terminator-FRT Kan FRT::*pstS* allele into MG1655. The P1 lysate was obtained courtesy of Nate Lord (Paulsson Lab, Harvard Medical School).

Strain BO56 was built by tagging the SprE gene with 2x mNeonGreen (also referred to as tandem mNeonGreen (tdNG)) fused to the AP tag. The SprE gene was modified at the endogenous chromosomal locus using the λRed method. Plasmid pDML199 was used as the PCR template for amplifying the integration cassette with 300-bp upstream and downstream homologies (to increase transformation and targeting efficiency). The 300-bp upstream and downstream homology regions were PCR amplified from genomic DNA (MC400) using primers DML_P674_F1 and DML_P675_R1 and primers DML_P676_F2 and DML_P677_R2, respectively, and PCR stitched to the integration cassette. Correct chromosomal integration was confirmed with colony PCR using primers DHL_P80_F and DML_P682_R. The FRT-flanked Kan marker was excised using pCP20, following removal of the plasmid by growing the cells at the non-permissive temperature in rich media. The *galK*::P_{lac}-CFP-Amp allele was then P1 transduced from Wc.

Strain DHL51 was built by P1 transducing the *rpoS*::Tn10(tet) allele into MC4100. The P1 lysate was obtained from Celeste Peterson (Suffolk University).

Strain DHL222 was built by integrating the RpoS750-Venus degradation reporter into the *phoA* locus using the λRed method following a standard protocol[8]. The P_{rpoS}-*rpoS750*-Venus-T1 terminator-FRT Kan FRT integration cassette was PCR amplified from pDHL39 with primers DHL_P93_F and DHL_P94_R. The upstream flank of the integration site was PCR verified with primers DHL_P101_F and DHL_P46_R; and the downstream flank of the integration site was verified with primers DHL_P47_F and DHL_P104_R.

Strain DHL241 was built by P1 transducing the *sprE*::Tn10(tet) allele into MC4100. The P1 lysate was obtained from Celeste Peterson (Suffolk University).

Strain DHL307 was built by PCR amplifying the 3xFLAG-FRT Kan FRT casette from pDHL229 with primers DHL_P107_F and DHL_P108_R. The purified integration cassette was integrated into strain DHL193 as previously described[8]. Tagging of *sprE* with the 3xFLAG tag was PCR verified with primers DHL_P120_F and DHL_P121_R.

Strain DHL331 is identical to strain DHL307 except that the FRT-flanked Kan marker was removed with pCP20.

DHL339 was built by P1 transducing the *rpoS*::Kan allele from CNP77 into MC4100.

Strain DHL394 was constructed by PCR amplifying the Venus-T1 terminator-FRT Kan FRT cassette from pDHL146 with primers DHL_P168_F and DHL_P169_R and integrating the cassette into strain DHL193 as previously described[8]. The upstream flank of the integration scar was PCR verified with primers DHL_P120_F and

DHL_P79_R, whereas the downstream flank of the integration scar was verified with primers DHL_P80_F and DHL_P81_R.

Strain DHL399 is identical to strain DHL394 except that the *sprE*-Venus-T1 terminator-FRT Kan FRT cassette was P1 transduced from DHL193 (i.e. after the λRed integration) into "fresh" MC4100 cells.

Strain DHL812 is identical to strain DHL399 except that the FRT-flanked Kan marker was excised using pCP20.

Strain DHL934 was built by transforming plasmid pDHL917 into MC4100.

Strain GL15 was built by P1 transducing the *galK*::P_{lac}-CFP-Amp allele from Wc into strain DHL222 after the FRT-flanked Kan marker was removed from DHL222 with pCP20.

Strain GL19 was built by P1 transducing the *galK*::P_{lac}-CFP-Amp allele from the Wc strain into MC4100.

Strain GL45 was built tagging the SprE gene with mNeonGreen at the native locus using the λRed method (see above). The integration cassette was PCR amplified using plasmid pDML22 as a template and primers DHL_P168_F and DHL_P169_R. The FRT-flanked Kan marker was removed with pCP20, and then the *galK*::P_{lac}-CFP-Amp allele was P1 transduced from Wc into this strain.

Strain GL64 was built by first P1 transducing the *rpoS*::Kan allele from JW5431 (KEIO collection) into strain GL45. Second, the FRT-flanked Kan marker was removed with pCP20 following P1 transduction of the *galK*::P_{lac}-CFP-Amp allele. The P1 lysate was obtained from the Wc strain.

Plasmid construction

All plasmids that were used in this study are listed in **Supplementary Table 3**.

The plasmid construction was performed using traditional 'sticky-end' cloning or isothermal assembly[9]. Analytical restriction digests or PCR were used to verify the plasmid construction. DNA sequencing was used to verify all cloning steps that involved PCR amplification. Phusion (Finnzymes), Vent (NEB), and Q5 (NEB) polymerases were used for the PCR reactions. The restriction enzymes were purchased from NEB and used according to the instructions provided by the manufacture.

pDHL16 was built by amplifying the full-length *rpoS* promoter (P_{rpoS}) and the first 750 base pairs of the *rpoS* open-reading frame (*rpoS*750) from genomic DNA (MC4100) with primers DHL_P13_F and DHL_P14_R. The full-length *rpoS* promoter includes the preceding *nlpD* gene including the *nlpD* promoter. The PCR product was

gel purified, digested with EcoRI and SacI, and then ligated in pUC19, which was cut with the same restriction enzymes and also gel purified.

pDHL17 was built by amplifying Venus and the T1 terminator from pPM1 (courtesy of Per Malkus) with primers DHL_P15_F and DHL_P16_R. The PCR product was digested with SacI and XmaI, gel purified, and ligated into pUC19, which was also digested with SacI and XmaI followed by gel purification.

pDHL23 was built by digesting pDHL17 with SacI and XmaI. The liberated Venus-T1 terminator fragment was then gel purified and subcloned into pDHL16, which was also digested with SacI and XmaI followed by gel purification.

pDHL39 was built by digesting pDHL23 with EcoRI and XmaI. The liberated P_{rpoS}-*rpoS*750-Venus-T1 terminator fragment was then gel purified and ligated into EcoRI/XmaI-cut pDHL19.

pDHL229 was constructed by PCR amplifying the 3xFLAG tag from plasmid pSUB11[10] with primers DHL_P105_F and DHL_P106_R. The PCR fragment was then cut with SacI and XmaI, gel purified and ligated into pDHL19, which was digested with SacI and XmaI and also gel purified.

pDHL655 was built in two steps. First, mGFPmut3 was amplified from pDHL580 using primers DHL_P21_F and DHL_P315_R. The PCR product was digested with SacI and XmaI, and then gel extracted. The purified PCR product was ligated into pDHL19, which was cut with the same enzymes and also gel extracted. Secondly, the resulting plasmid was digested with XmaI, gel extracted, and an oligo site, which encodes the 15-amino-acid long acceptor peptide (AP) tag[11], was inserted into the vector backbone. The oligo site was made by annealing primers DHL_P316_F and DHL_P317_R following a standard protocol (see e.g. https://www.addgene.org/plasmid-protocols/annealed-oligo-cloning/).

pDHL694 was built by PCR amplifying Dronpa from pDHL583 with primers DHL_P255_F and DHL_P348_R. The PCR product was digested with SacI and XmaI, gel purified, and ligated into pDHL655, which was also cut with the restriction enzymes SacI and XmaI and then gel purified.

pDML830 was constructed in two steps. Frist, Zif268 was PCR amplified from a plasmid template that encodes Zif268 coding sequence (courtesy of the Silver lab, Harvard Medical School). The PCR product was then digested with BamHI and HindIII, gel extracted, and ligated into pPM16, which was cut with the same enzymes and also gel purified. Secondly, the resulting plasmid was opened with the restriction enzymes HindIII and XbaI, and gel extracted. Primers DHL397_F and DHL_398_R were used to make an oligo site that encodes the AP tag. The oligo site was then inserted into the opened vector.

pDHL917 was built by first digesting pDHL830 with BamHI and HindIII, which cuts out the DNA sequence that encodes the Zif268. The vector was then gel purified. The mEos2 coding sequence was PCR-amplified with primers DHL_P485_F and DHL_P486_R using pDHL844 as a template. mEos2 was then inserted into the BamHI/HindIII-digested pDHL830 vector with isothermal assembly.

pDML22 was built by PCR amplifying mNeonGreen with primers DML_P118_F and DML_P119_R from pUC57-Kan-mNeonGreen. The plasmid was a gift from the Lindquist lab (Whitehead Institute at MIT). The PCR product was inserted into vector pDHL580, which was cut with BlpI and XmaI, using isothermal assembly.

pDML199 was built by PCR amplifying the first mNeonGreen from plasmid pDML48 using primers DML_P637_F and DML_P638_R and the second mNeonGreen from plasmid pDML152 using primers DML_P635_F and DML_P636_R. The second mNeonGreen is yeast codon-optimized and has hence no local sequence identity with the first mNeonGreen (though the global sequence identity is ~78%). Avoiding sequence homology is important to prevent recombination between the two mNeonGreen parts, which would reduce the tandem mNeonGreen to a single mNeonGreen. The PCR-amplified mNeonGreens were then inserted with isothermal assembly into pDHL694, which was digested with SacI and XmaI and also gel purified. Plasmids pDML48 and pDML152 were generous gifts from the Lindquist lab (Whitehead Institute at MIT, Cambridge, MA, USA).

All plasmids and the corresponding vector maps are available upon request.

Primers

The primers used in this study are listed in **Supplementary Table 4**. The Primers were purchased from Integrated DNA Technologies, Inc. (Coralville, IA, USA).

Supplementary References

1. Montero Llopis, P., Sliusarenko, O., Heinritz, J. & Jacobs-Wagner, C. In vivo biochemistry in bacterial cells using FRAP: insight into the translation cycle. *Biophys. J.* **103,** 1848–1859 (2012).
2. Kuhlman, T. E. & Cox, E. C. Gene location and DNA density determine transcription factor distributions in Escherichia coli. *Molecular Systems Biology* **8,** 1–13 (2012).
3. Pertsinidis, A., Zhang, Y. & Chu, S. Subnanometre single-molecule localization, registration and distance measurements. *Nature* **466,** 647–651 (2010).
4. Bakshi, S., Bratton, B. P. & Weisshaar, J. C. Subdiffraction-Limit Study of Kaede Diffusion and Spatial Distributionin Live Escherichia coli. *Biophysj* **101,** 2535–2544 (2011).
5. Dill, K. A., Ghosh, K. & Schmit, J. D. Inaugural Article: Physical limits of cells and proteomes. *Proceedings of the National Academy of Sciences* **108,** 17876–17882 (2011).
6. Parry, B. R. *et al.* The Bacterial Cytoplasm Has Glass-like Properties and Is Fluidized by Metabolic Activity. *CELL* **156,** 183–194 (2013).
7. Shaner, N. C. *et al.* A bright monomeric green fluorescent protein derived from Branchiostoma lanceolatum. *Nat. Methods* **10,** 407–409 (2013).
8. Landgraf, D., Okumus, B., Chien, P., Baker, T. A. & Paulsson, J. Segregation of molecules at cell division reveals native protein localization. *Nat. Methods* **9,** 480–482 (2012).
9. Gibson, D. G. *et al.* Enzymatic assembly of DNA molecules up to several hundred kilobases. *Nat. Methods* **6,** 343–345 (2009).
10. Uzzau, S., Figueroa-Bossi, N., Rubino, S. & Bossi, L. Epitope tagging of chromosomal genes in Salmonella. *Proc. Natl. Acad. Sci. U.S.A.* **98,** 15264–15269 (2001).
11. Chen, I., Howarth, M., Lin, W. & Ting, A. Y. Site-specific labeling of cell surface proteins with biophysical probes using biotin ligase. *Nat. Methods* **2,** 99–104 (2005).
12. Jensen, K. F. The Escherichia coli K-12 'wild types' W3110 and MG1655 have an rph frameshift mutation that leads to pyrimidine starvation due to low pyrE expression levels. (1993).
13. Casadaban, M. J. Transposition and fusion of the lac genes to selected promoters in Escherichia coli using bacteriophage lambda and Mu. *Journal of Molecular Biology* **104,** 541–555 (1976).
14. Hegreness, M., Shoresh, N., Hartl, D. & Kishony, R. An equivalence principle for the incorporation of favorable mutations in asexual populations. *Science* **311,** 1615–1617 (2006).
15. Datsenko, K. A. & Wanner, B. L. One-step inactivation of chromosomal genes in Escherichia coli K-12 using PCR products. *Proc. Natl. Acad. Sci. U.S.A.* **97,** 6640–6645 (2000).
